# Establishment of a functional system for recombinant production of secreted proteins at 50 °C in the thermophilic *Bacillus methanolicus*

**DOI:** 10.1186/s12934-020-01409-x

**Published:** 2020-07-28

**Authors:** Marta Irla, Eivind B. Drejer, Trygve Brautaset, Sigrid Hakvåg

**Affiliations:** Department of Biotechnology and Food Sciences, Norwegian University of Science and Technology (NTNU), Trondheim, Norway

**Keywords:** Signal peptide library, Secretion, sfGFP, Thermophile, α-amylase

## Abstract

**Background:**

The suitability of bacteria as microbial cell factories is dependent on several factors such as price of feedstock, product range, production yield and ease of downstream processing. The facultative methylotroph *Bacillus methanolicus* is gaining interest as a thermophilic cell factory for production of value-added products from methanol. The aim of this study was to expand the capabilities of *B.* *methanolicus* as a microbial cell factory by establishing a system for secretion of recombinant proteins.

**Results:**

Native and heterologous signal peptides were tested for secretion of α-amylases and proteases, and we have established the use of the thermostable superfolder green fluorescent protein (sfGFP) as a valuable reporter protein in *B.* *methanolicus*. We demonstrated functional production and secretion of recombinant proteases, α-amylases and sfGFP in *B.* *methanolicus* MGA3 at 50 °C and showed that the choice of signal peptide for optimal secretion efficiency varies between proteins. In addition, we showed that heterologous production and secretion of α-amylase from *Geobacillus stearothermophilus* enables *B.* *methanolicus* to grow in minimal medium with starch as the sole carbon source. An in silico signal peptide library consisting of 169 predicted peptides from *B.* *methanolicus* was generated and will be useful for future studies, but was not experimentally investigated any further here.

**Conclusion:**

A functional system for recombinant production of secreted proteins at 50 °C has been established in the thermophilic *B. methanolicus*. In addition, an in silico signal peptide library has been generated, that together with the tools and knowledge presented in this work will be useful for further development of *B.* *methanolicus* as a host for recombinant protein production and secretion at 50 °C.

## Background

### Thermophilic bacteria as hosts for recombinant protein production

Thermophilic bacteria are gaining interest both as sources of heat resistant enzymes, but also as alternative production hosts for thermostable proteins [1–4]. Heat resistant enzymes have innate advantages over their mesophilic counterparts, including increased temperature stability and resistance to proteolytic cleavage by proteases [5]. Consequently, thermophilic species such as *Bacillus licheniformis* and *Geobacillus stearothermophilus* are utilized as donors of industrially important enzymes like α-amylases and proteases, which are extensively used for detergent production [6, 7]. Many of these enzymes can fold properly at temperatures 60 °C below their physiological conditions, and have the same stability, catalytic or structural properties as those purified from the native organism, but attempts to express hyperthermophilic proteins in *E. coli* revealed that 50% of the proteins were found in the insoluble fraction of cell lysates [8, 9]. It could therefore be advantageous to explore thermophilic hosts such as *B. methanolicus* as overproducers of these proteins at elevated temperatures.

Thermophilic bacteria can potentially be used as platforms for efficient functional screening of thermostable enzymes at elevated temperatures [1, 3]. The benefits of utilizing thermophilic hosts in bioprocesses include decreased risk of contamination of fermentation cultures and lower cooling costs compared to mesophilic hosts [10, 11]. It has also been suggested that some thermostable enzymes need high temperatures (and consequently thermophilic hosts) for proper expression and folding [12]. Many thermophilic organisms grow slowly and have low biomass productivities, making them poor choices as hosts for industrial production of proteins, however *B. methanolicus* can reach specific growth rates of 0.46 h^−1^, making it a good choice as a thermophilic host for protein production [13, 14].

Among thermophiles, thermophilic Bacillaceae possess several advantages for recombinant production of secreted thermostable enzymes. Most Bacillaceae strains are nonpathogenic, and more importantly, are particularly suited to secrete recombinant proteins [15, 16]. Members of the *Bacillus* genus have been shown to secrete a large number of proteins into their environment, among them α-amylases and proteases, mainly through the general secretory pathway (Sec) which is the most commonly used secretion pathway in Gram-positive bacteria for biotechnological purposes [17, 18]. A distinguishing feature of protein secretion through the Sec pathway is that the folding takes place in the oxidizing extra-cytoplasmic environment, leading to fewer folding issues for some proteins such as those containing disulfide bridges [18]. In *E. coli*, overexpression of proteins often results in the formation of inclusion bodies, hampering the purification process of correctly folded active proteins, and hence secretion to the extracellular medium is an important motivation for selecting Gram-positive bacteria as hosts for production of heterologous proteins [18].

Gram-positive bacteria are considered desirable hosts for recombinant protein secretion due to their cell envelope structure, which ideally results in direct release of small, secreted proteins into the culture supernatant. Several Gram-positive species such as *B. subtilis* and *B. licheniformis* are able to secrete native enzymes in high titers, and have therefore been considered as promising host candidates for recombinant protein secretion [19–21]. The innate permeability of the outer cell wall in Gram-positive bacteria is estimated to allow free passage of globular proteins up to 25 kDa in size, and it is believed that the negatively charged environment in the extra-cytoplasmic space can recruit more cations such as Ca^2+^ which are important cofactors for enzymes like amylases [22, 23]. Recombinant production of proteins in the cytoplasm, or by secretion to the periplasm in Gram-negative species often results in formation of inclusion bodies, and recovering proteins from these inclusion bodies can be difficult and expensive [18, 24]. Additionally, Gram-negative production hosts can have difficult-to-remove endotoxins as part of their cell wall, making protein purification more complicated and expensive [24]. The cell envelope structure in Gram-positive bacteria avoids these two issues, as the cell wall lacks endotoxins, and secretion across the cytoplasmic membrane places proteins in the extracellular environment where they can fold correctly [18, 25].

The factors that negatively affect protein secretion most prominently in Gram-positive bacteria are limited availability of chaperones specific for cytoplasmic protein secretion and the activity of extra-cytoplasmic quality control and feeding proteases [20, 26–30]. Despite these challenges, several *Bacillus* species are successfully used for overproduction of industrially desirable enzymes, with high titers achieved for natively secreted proteins [31]. The secretion of some of these enzymes, such as amylases, is influenced by PrsA, an extra-cytoplasmic lipoprotein chaperone which is essential for growth in *B. subtilis* [32, 33]. However, most secreted proteins are unaffected by the presence or absence of PrsA. Instead, it is believed that many natively secreted proteins have evolved to escape degradation by proofreading and feeding proteases by rapid folding after translocation to ensure that the vulnerable target sites are buried within the proteins [26]. Additionally, pro-peptide regions situated between the signal peptide and the mature protein sequence are found in many of these proteins, and are believed to act as intrinsic folding chaperones [34].

Efforts in strain engineering for efficient protein secretion in *B. subtilis* have been aimed at chromosomal deletions of quality control and feeding proteases, as well as increasing the expression levels of secretion chaperones that stabilize pre-proteins in a secretion-competent state in the cytoplasm, leading to lowered secretion stress and fewer inclusion bodies [20, 35, 36]. Finally, for proteins dependent on PrsA for folding, its overexpression has been shown to increase titers of secreted proteins significantly [19, 35, 37, 38]. A gene encoding PrsA, is also present in the genome of *B. methanolicus* strain MGA3 (BMMGA3_04045).

Extracellular secretion allows enzymes to be functionally assayed directly in liquid or on solid medium, yields simplified product recovery, and facilitates downstream processing [3, 18, 39]. In *E. coli*, secretion of recombinant proteins can generate titers in the scale of 5–10 g/L for several proteins with optimized production processes [40]. Furthermore, comparisons between secretion and cytoplasmic titers for proteins heterologously produced in *Lactococcus lactis* show that secretion titers are two to tenfold higher than cytoplasmic ones [41]. Recent progress has been made in developing replicons, promoters (constitutive and inducible), selection markers and other tools for genetic engineering of thermophilic Bacillaceae [42]. However, it is still necessary to develop new tools for establishing these bacteria as feasible hosts for recombinant production of secreted proteins.

### *Bacillus methanolicus* as a host for industrial biotechnology at 50 °C

The thermophilic *Bacillus methanolicus* is a promising candidate to become an industrial workhorse for methanol-based production of value-added compounds such as amino acids and their derivatives at 50 °C, with l-glutamate and l-lysine reaching titers of 59 g/L and 65 g/L, respectively, and cadaverine and γ-aminobutyric acid (GABA) reaching titers of 11 g/L and 9 g/L, respectively [14, 43–45]. *B.* *methanolicus* can utilize methanol as its sole carbon and energy source, but is as a facultative methylotroph also able to utilize other carbons sources, including mannitol and glucose [46–48].

Extensive progress has been made with regards to characterization of the genetics, physiology and metabolism of *B.* *methanolicus*, with a sequenced genome, mapped transcriptome and a thorough investigation of its proteome and metabolome being available [49–53]. The genetic toolbox for *B.* *methanolicus* has also expanded in the last years, including new gene expression systems and a system for gene silencing [54, 55]. So far, *B.* *methanolicus* has mainly been used for production of small molecules, and in order to establish recombinant protein secretion in *B.* *methanolicus* at 50 °C, reporter proteins functionally expressed at elevated temperatures are needed [14, 44, 45, 54, 56, 57]. So far, two different mesophilic reporter proteins, GFPuv and a *Streptomyces griseus*-derived α-amylase have been recombinantly expressed in *B.* *methanolicus;* however the temperatures of these cultivations were reduced to 37–40 °C to obtain functional proteins, and production yields were not quantified in either case due to the proof-of-concept nature of the studies. Additionally, GFPuv was in this case not fused to a signal peptide and was expected solely to be produced intracellularly, and consequently the fluorescence was only measured for the cell pellets [54, 56].

### Signal peptides for recombinant protein secretion

Secretion of proteins in prokaryotes is an important process which allows modification and scavenging of resources from the extracellular environment in addition to modification of cell structures such as cell walls or membranes [58, 59]. As an example, amylases are secreted into the environment in order to catalyze hydrolysis of polysaccharides into simple sugars which are easily metabolized by microorganisms. Secretion is guided by signal sequences at the N-terminal end of the native proteins, and these sequences can thus be fused to recombinant target proteins for secretion. Secretion of proteins into the extracellular environment can be an advantage for simplified downstream processing of protein products because purification from cultivation broth is less demanding than extraction from cell biomass which requires cell lysis and extensive isolation processes [60]. It has been demonstrated that fusion of signal peptides to different proteins can result in hampering of secretion and thermodynamic destabilization, reduced activity and increased tendency to aggregate of the target protein. The in-depth inspection of interactions between signal peptides fused to different proteins suggests that for each signal peptide-protein fusion, the effect on protein thermodynamic stability is unpredictable and dependent on both components of the structure and their interplay [61–63]. This leads to a need for screening large signal peptide libraries in order to identify optimal combinations of signal peptides and proteins when establishing secretion of heterologous proteins [61, 64, 65]. Signal peptide libraries for *B.* *subtilis* are now commercially available from suppliers such as TaKaRa Bio [66, 67]. Thanks to the widespread availability of genome sequences in modern biotechnology, a common initial strategy to identify putative signal peptides for in vivo screening is to use signal peptide prediction algorithms on the genomes of organisms of interest [68]. This can be done with sequence prediction software such as SignalP, Phobious, PrediSi, TatP, Tatfind and PRED-TAT [69–75]. While this approach can be used to identify native signal peptide sequences, it is likely that a knowledge-based approach can improve the effectiveness of recombinant protein secretion. Strategies for signal peptide optimization include random or targeted mutagenesis combined with screening for increased secretion activity, or screening of signal peptide libraries containing peptides from multiple species, and approaches such as these can incur extensive workloads depending on the ease of the screening process [76].

The goal of this study was to establish a system for recombinant production of secreted proteins under thermophilic conditions in *B.* *methanolicus.* To enable this, thermostable reporter proteins and functional signal peptides were needed. Thermostable α-amylases and proteases were used in this study, both due to the presence of native signal peptides, and their easily assayed activities. In our study, thermostable sfGFP was established as a reporter protein in *B.* *methanolicus* and applied to investigate the impact of the signal peptides on the ratio of intracellular to extracellular protein. Our results expand the potential applications of *B.* *methanolicus* beyond production of amino acids and their derivatives and into the production and secretion of recombinant proteins at elevated temperatures.

## Results

### Selection of candidate α-amylases and proteases for recombinant production and secretion in *Bacillus methanolicus*

In this study, α-amylases and proteases were chosen as two potential groups of reporter proteins since they are likely to posess native signal sequences, are easy to assay and industrially relevant.

To select candidate protease and amylase genes, the genomes of *B.* *methanolicus* and three taxonomically closely related species were searched for genes encoding α-amylases and proteases. The genome of *B.* *methanolicus* MGA3 was found to contain two genes putatively coding for α-amylases (BMMGA3_04340 and BMMGA3_04345), and BMMGA3_04345 was selected as a reporter protein in this study. The genomes of the *B.* *subtilis* 168, *B.* *licheniformis* MW3 and *G.* *stearothermophilus* 10 were screened for α-amylase and protease encoding genes with native signal peptides. Two criteria were emphasized when choosing the target proteins: thermostability and significant primary sequence heterogeneity between isoenzymes. As shown in Table 1, the α-amylases from *B.* *licheniformis* (AmyL), *B.* *subtilis* (AmyE) and *G.* *stearothermophilus* (AmyS) selected for this study are reported to have temperature optima at 90 °C, 50 °C and 65 °C, respectively [77–79], while *apr*-encoded subtilisin from *B.* *licheniformis* has a temperature optimum at 50 °C, and the *aprE*-encoded protease from *B.* *subtilis* 168 has a homolog from *B.* *subtilis* A26 (91.34% identity) whose optimum temperature is at 60 °C [80, 81]. Even though the α-amylases chosen for this study were derived from closely related organisms, their amino acid sequences differ substantially from the *B.* *methanolicus*-derived Amy, with sequence similarities of 22.47%, 27.62% and 24.86% for AmyS, AmyE and AmyL, respectively, ensuring that different signal peptides and also different model proteins were tested in *B.* *methanolicus*. The evolutionary relationship of the α-amylase primary sequences from the different Bacillaceae is shown in Additional file 1: Figure S1.

**Table 1:** Proteins with native signal peptides used in this study

Product	Native organism	Protein		Signal peptide		
		Accession number	Temperature optimum^a^ (°C)	SignalP-5.0 (probability)	PrediSI (score)	Cleavage site^c^ (amino acid)
α-Amylase				Sec/SPI		
*amyL*	*B.* *licheniformis*	BL00499	90	0.9792	0.9872	28/29, 28
*amyE*	*B.* *subtilis*	BSU03040	50	0.9718	0.6027	29, 33/34
*amyS*	*G.* *stearothermophilus*	GT50_07800	65	0.9807	1.0000	34/35, 34
* amy*	*B.* *methanolicus*	BMMGA3_04345	ND	0.9931	0.6850	23/24, 24
Protease						
*apr*	*B.* *licheniformis*	BLI_RS05490	50	0.9935	0.9128	29/30, 29
*aprE*	*B.* *subtilis*	GP2222_11200	60^b^	0.9929	0.8976	29/30, 29
*apr*	*G.* *stearothermophilus*	GT50_00190	NC	0.6871	0.6283	30/31, 30

Signal peptides were analyzed by SignalP-5.0 and PrediSI [69, 71]

*ND* not determined, *NC* not characterized. To the best of the authors’ knowledge

^a^ Temperature optimum of proteins previously determined. See text for details and references

^b^ Temperature optimum for homolog protease-gene in *B.* *subtilis* A26

^c^ As predicted by SignalP-5.0 and PrediSI, respectively [69, 71]

The selected reporter proteins were then analysed with SignalP-5.0 and PrediSI and were all predicted to possess signal peptides of the Sec/SPI-type, which are secretory signal peptides transported by the Sec translocon and cleaved by Signal Peptidase I [69, 71]. SignalP-5.0 and PrediSI use different methods to predict signal peptides; SignalP-5.0 uses a deep neural network-based method combined with conditional random field classification and optimized transfer learning, and Predisi uses positional weighted matrices. The predicted probability for the proteins to contain signal peptides are listed in Table 1 together with the score obtained by PrediSI-analysis, where a score greater than 0.5 means that the examined sequence very likely contains a signal peptide. Similarly, an α-amylase from *Streptomyces griseus* IMRU3570 which was previously heterologously produced and secreted in *B.* *methanolicus* [54], was analysed and shown to have SignalP-5.0 and PrediSI values of 0.8683 and 1.0000 respectively.

### Functional secretion of recombinant proteases and α-amylases in *B.* *methanolicus* at 50 °C

The selected protease and amylase genes (Table 1) were initially cloned into the vector pBV2xp, under control of the inducible xylose promoter, xp, and the resulting plasmids were introduced into the *B.* *methanolicus* wild type strain MGA3 (Table 2). The inducible xp promoter was chosen to enable the regulation of expression levels, and because when fully induced, the xp promoter is the strongest known promoter for *B.* *methanolicus* [54]. The recombinant strains were induced with 10 g/L xylose, in order to maximize expression of the reporter genes. Formation of clearing zones around cells in plate assays indicates hydrolysis of casein due to the activity of secreted protease. The activity of a secreted recombinant protease could be detected in plate assays after 24 h for the strain spPBs-aprBs, carrying a protease gene and its native signal peptide from *B.* *subtilis* (Additional file 1: Figure S2a). By prolonging the incubation time (to 36 h), some protease activity was also detected for the strain spBl-aprBl (protease gene and signal peptide derived from *B.* *licheniformis*) (Additional file 1: Figure S2b). The promising results from the protease secreting strains will be further explored in future studies. We choose, however, to present here the initial investigation into protease secretion in *B. methanolicus* MGA3 to demonstrate the versatility of this strain for secretion of industrially relevant proteins.

**Table 2:** Strains and plasmids used in this study

Strain	Description		Reference
*Escherichia coli* DH5α		General cloning host, F-*thi*-1 *endA*1 *hsdR*17(r-, m-) *supE*44 _*lacU*169 (_80*lacZ*_M15) *recA*1 *gyrA*96 *relA*1	Stratagene/Genomics Agilent
*Bacillus methanolicus* MGA3		Wild type strain	ATCC53907
*B. licheniformis* MW3		*Bacillus licheniformis* DSM13 (*ΔhsdR1*, *ΔhsdR2*)	[100]
*B. subtilis* 168		Wild type strain	ATCC23857
*Geobacillus stearothermophilus* 10		Wild type strain	DSM13240

Plasmid	Strain abbreviation^a^	Description	Reference
pBV2xp	EV	Km^R^ and Ap^R^; pHCMC04 derivative, gene expression under the control of the inducible xylose promoter from *B.* *megaterium,* theta replicating	[83]
Protease gene carrying strains/plasmids			
pBV2xp-*aprBl*	spPBl-aprBl	Km^R^ and Ap^R^; pBV2xp derivative for expression of *apr* from *B.* *licheniformis* with native signal peptide (locus tag BLI_RS05490)	This study
pBV2xp-*aprBs*	spPBs-aprBs	Km^R^ and Ap^R^; pBV2xp derivative for expression of *aprE* from *B.* *subtilis* with native signal peptide (locus tag GP2222_11200)	This study
pBV2xp-*aprGs*	spPGs-aprGs	Km^R^ and Ap^R^; pBV2xp derivative for expression of *aprGs* from *G.* *stearothermophilus* with native signal peptide (locus tag GT50_00190)	This study
α-amylase gene carrying strains/plasmids			
pBV2xp-spBl-*amyBl*	spBl-amyBl	Km^R^ and Ap^R^; pBV2xp derivative for expression of *amyL* from *B.* *licheniformis* with native signal peptide (locus tag BL00499)	This study
pBV2xp-spBs-*amyBs*	spBs-amyBs	Km^R^ and Ap^R^; pBV2xp derivative for expression of *amyE* from *B.* *subtilis* with native signal peptide (locus tag BSU03040)	This study
pBVxp-spGs-*amyGs*	spGs-amyGs	Km^R^ and Ap^R^; pBV2xp derivative for expression of *amyS* from *G.* *stearothermophilus* with native signal peptide (locus tag GT50_07800)	This study
pBV2xp-spBm-*amyBm*	spBm-amyBm	Km^R^ and Ap^R^; pBV2xp derivative for expression of *amy* from *B.* *methanolicus* with native signal peptide (locus tag BMMGA3_04345)	This study
pBV2xp-spGs-*amyBs*	spGs-amyBs	Km^R^ and Ap^R^; pBV2xp derivative for expression of *amyE* from *B.* *subtilis* with α-amylase signal peptide from *G.* *stearothermophilus*	This study
pBVxp-spGs-*amyBl*	spGs-amyBl	Km^R^ and Ap^R^; pBV2xp derivative for expression of *amyL* from *B.* *licheniformis* with α-amylase signal peptide from *G.* *stearothermophilus*	This study
sfGFP gene carrying strains/plasmids			
sfGFP-pBAD		Am^R^; pBAD/His derivative for expression of *sfGFP* under the control of P_*araBAD*_	sfGFP-pBAD was a gift from Michael Davidson and Geoffrey Waldo (Addgene plasmid # 54519) [82]
pBV2xp-*sfGFP*	sp0-sfGFP	Km^R^ and Ap^R^; pBV2xp derivative for expression of *sfGFP* from sfGFP-pBAD under control of the *xp* promoter	This study
pBV2xp-spGs-*sfGFP*	spGs-sfGFP	Km^R^ and Ap^R^; pBV2xp-*sfGFP* derivative with amylase signal peptide from *G.* *stearothermophilus*	This study
pBV2xp-spBl-*sfGFP*	spBl-sfGFP	Km^R^ and Ap^R^; pBV2xp-*sfGFP* derivative with amylase signal peptide from *B.* *licheniformis*	This study
pBV2xp-spBs-*sfGFP*	spBs-sfGFP	Km^R^ and Ap^R^; pBV2xp-*sfGFP* derivative with amylase signal peptide from *B.* *subtilis*	This study
pBV2xp-spBm-*sfGFP*	spBm-sfGFP	Km^R^ and Ap^R^; pBV2xp-*sfGFP* derivative with amylase signal peptide from *B.* *methanolicus*	This study

^a^Abbreviation used for *B.* *methanolicus* strain MGA3 carrying the listed plasmid. spP: signal peptide preceding protease; sp: signal peptide preceding α-amylase. Example: Strain spBl-amyBl carries plasmid with (α-amylase) signal peptide from *B. licheniformis*, and α-amylase gene from *B. licheniformis*

*Km*^*R*^ kanamycin resistance marker, *Ap*^*R*^ ampicillin resistance marker

**Fig. 1:**
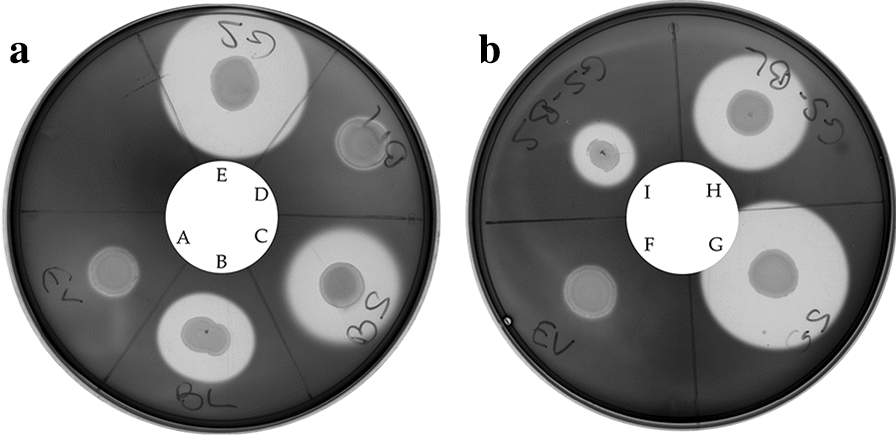
Detection of α-amylase activity from recombinant strains of *B.* *methanolicus* incubated at 50 °C. In the plate assay, hydrolysis of starch by α-amylase is seen as clearing zones around the colonies, visualized by addition of Lugol’s solution after 24 h incubation. Recombinant strains tested are: Plate **a**: EV (emtpy vector), used as control (A), spBl-amyBl (B), spBs-amyBs (C), spBm-amyBm (D) and spGs-amyGs (E). Plate **b**: Control strain EV (F), spGs-amyGs (G), spGs-amyBl (H) and spGs-amyBs (I)

In experiments involving α-amylase genes, altogether five strains were tested, namely an empty vector control strain carrying the pBV2xp plasmid (EV), and strains overexpressing α-amylase genes and their native signal peptides (sp), derived from *B.* *methanolicus* (spBm-amyBm) (homologous), *B.* *licheniformis* (spBl-amyBl), *B.* *subtilis* (spBs-amyBs) and *G.* *stearothermophilus* (spGs-amyGs). Activity of secreted α-amylases could be detected in plate assays, as shown in Fig. 1a (strains incubated at 50 °C). Clearing zones around cells indicate hydrolysis of starch due to the activity of secreted α-amylase from the recombinant strains spBs-amyBs, spBl-amyBl or spGs-amyGs (Fig. 1). To rule out that a temperature of 50 °C negatively affected secretion of active enzymes, the experiments were also performed at 37 °C, with similar results (Additional file 1: Figure S3). As mentioned before, the genome of *B.* *methanolicus* MGA3 contains two genes putatively encoding α-amylases, however, the sequence similarity to α-amylases from other bacilli is low, and detection of amylase activity from wild type strains of *B. methanolicus* is currently not reported. Hydrolysis of starch could not be detected from the control strain EV under these conditions and clearing zones were also not detected around the recombinant strain spBm-amyBm, overexpressing one of these putative α-amylases. The presence of extracellular α-amylase activity at 50 °C in plate assays demonstrates the potential of *B.* *methanolicus* as a host for heterologous production of secreted proteins under thermophilic conditions. Based on the results of α-amylases plate assays (visible halo for 3 out of 4 strains tested after 24 h), it was decided to further analyze the α-amylase-secreting strains in shake flask experiments.

**Fig. 2:**
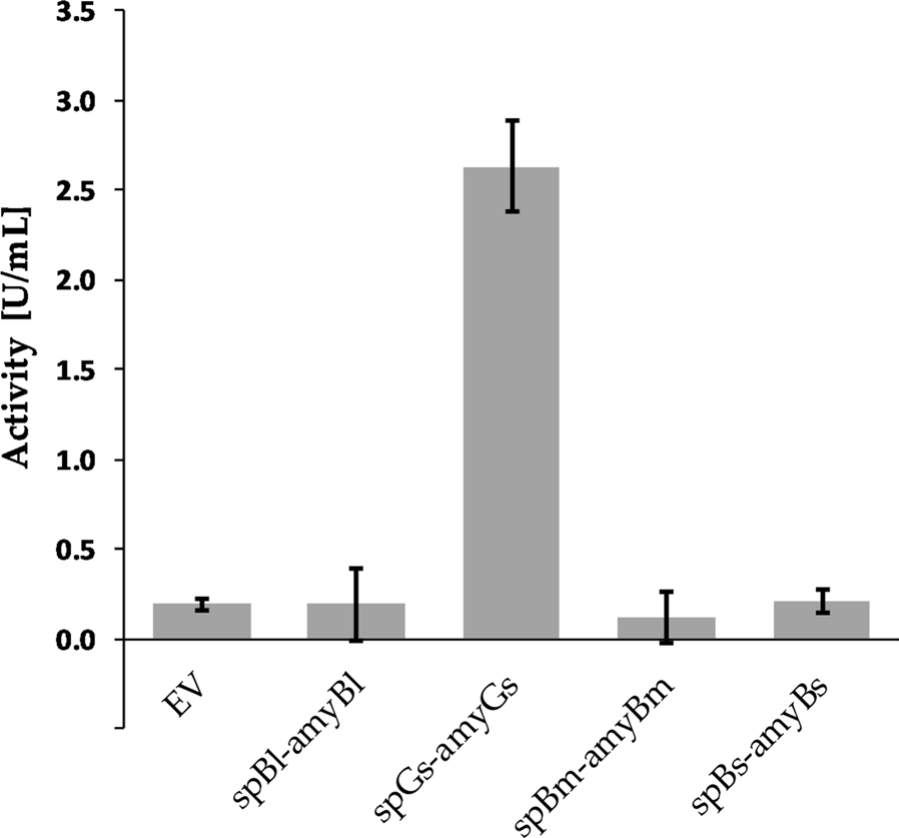
α-Amylase activity in culture supernatants of recombinant *B.* *methanolicus* MGA3 strains. The *B.* *methanolicus* MGA3 strains express α-amylase genes with native signal peptide from *B.* *licheniformis* (spBl-amyBl), *G.* *stearothermophilus* (spGs-amyGs), *B.* *subtilis* (spBs-amyBs) and *B.* *methanolicus* (spBm-amyBm). Strain carrying empty vector (EV) is used as control. Maximum and minimum measured values for triplicate shake flask cultures are presented

In the liquid assays, α-amylase activity could be detected in supernatants of strain spGs-amyGs (2.6 U/mL) as shown in Fig. 2, confirming that the functional enzyme was secreted when fused to the spGs signal peptide. Activity in the liquid culture supernatants was measured both as units per mL (see Fig. 2) and units per mg of total protein (results not shown), with coinciding relative results. Based on the available results, it cannot be determined whether the lack of detected activity in the cultivation broth from strains spBs-amyBs and spBl-amyBl (Fig. 2) despite halo formation in the plate assay (Fig. 1) was due to poorly functioning signal peptides, low expression level or the detection limits of the enzyme assay under these conditions. To investigate this further, the functional signal peptide from *G.* *stearothermophilus* (spGs) was used to replace the native signal peptides of the α-amylases from *B.* *subtilis* and *B.* *licheniformis.* The resulting strains spGs-amyBs and spGs-amyBl were then tested in plate assays and liquid culture assays.

### Substituting signal peptides does not result in increased α-amylase secretion

**Fig. 3:**
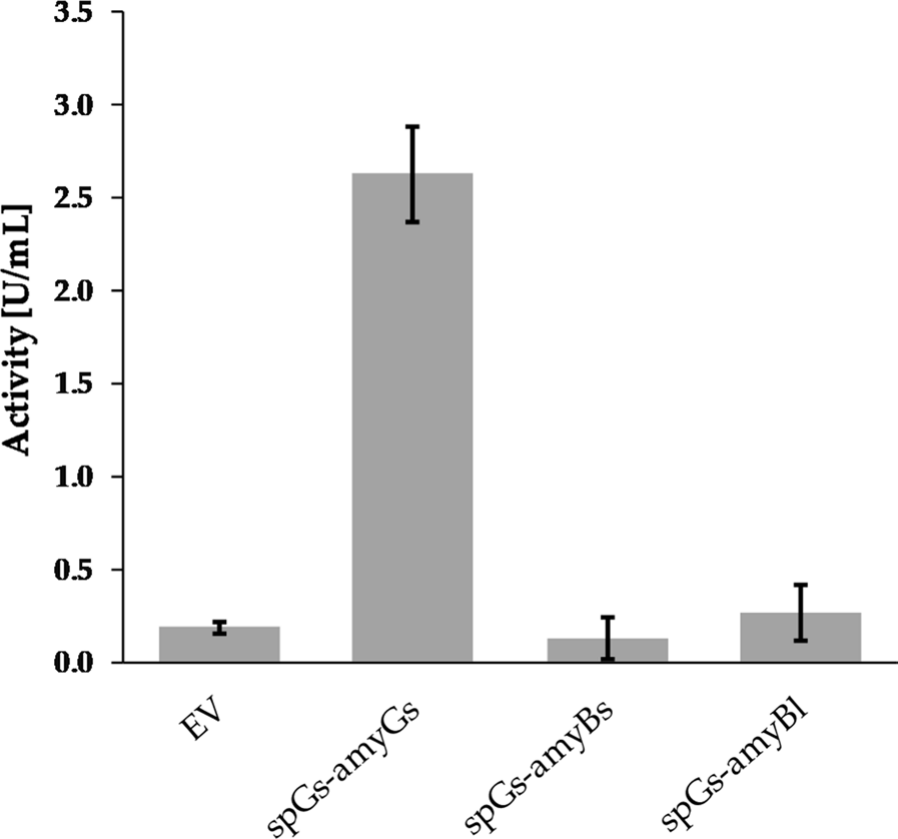
α-Amylase activity in culture supernatants of *B. methanolicus* MGA3 recombinant strains. The *B.* *methanolicus* MGA3 strains express signal peptide from *G.* *stearothermophilus* linked to α-amylase genes from *B.* *subtilis* (spGs-amyBs) or *B.* *licheniformis* (spGs-amyBl). Strain carrying empty vector (EV) is used as negative control. Strain spGs-amyGs carrying α-amylase gene from *G.* *stearothermophilus* and its native signal peptide is used as positive control. Maximum and minimum measured values for triplicate shake flask cultures are presented

Initial plate assays were performed with the strains spGs-amyBs and spGs-amyBl, and activity of secreted α-amylases could be detected at both 50 °C (Fig. 1b) and 37 °C (Additional file 1: Figure S3b) for both strains. Strains EV and spGs-amyGs were used as controls. Shake flask cultivations were then performed with the strains spGs-amyBs, spGs-amyBl and the spGs-amyGs reference strain, and α-amylase activity in the supernatants of the induced cultures was measured. As shown in Fig. 3, no increase in activity was detected from the strains spGs-amyBs and spGs-amyBl in comparison to the supernatants of strains producing α-amylases with their native signal peptides (spBs-amyBs and spBl-amyBl) (Fig. 2). It is, however, yet to be determined whether the lack of detectable α-amylase activities in the supernatants of strains spGs-amyBs and spGs-amyBl were caused by poor secretion, rather than by other factors affecting enzyme activity (e.g. expression levels). Additional secretion experiments were performed, in which one functional reporter protein (sfGFP) was fused to the different α-amylase signal peptides (see below).

### Recombinant expression of secreted sfGFPconfirms the functionality of heterologous signal peptides in *B. methanolicus*

Based on the results above, one common reporter protein was selected in order to distinguish functionality of the signal peptide-protein fusion from the activity of the expressed enzyme. The reporter must be functionally expressed, stable at the selected cultivation temperature, and preferably easily assayed. Fluorescent assays require no cell lysis, and enable straightforward detection of fluorescence from both pelleted cells and culture supernatants, allowing a more extensive analysis. The use of GFPuv has earlier been described for *B.* *methanolicus* in temperatures up to 40 °C [54, 56], however functional expression of a GFP by *B.* *methanolicus* at 50 °C has not been reported prior to this work.

**Fig. 4:**
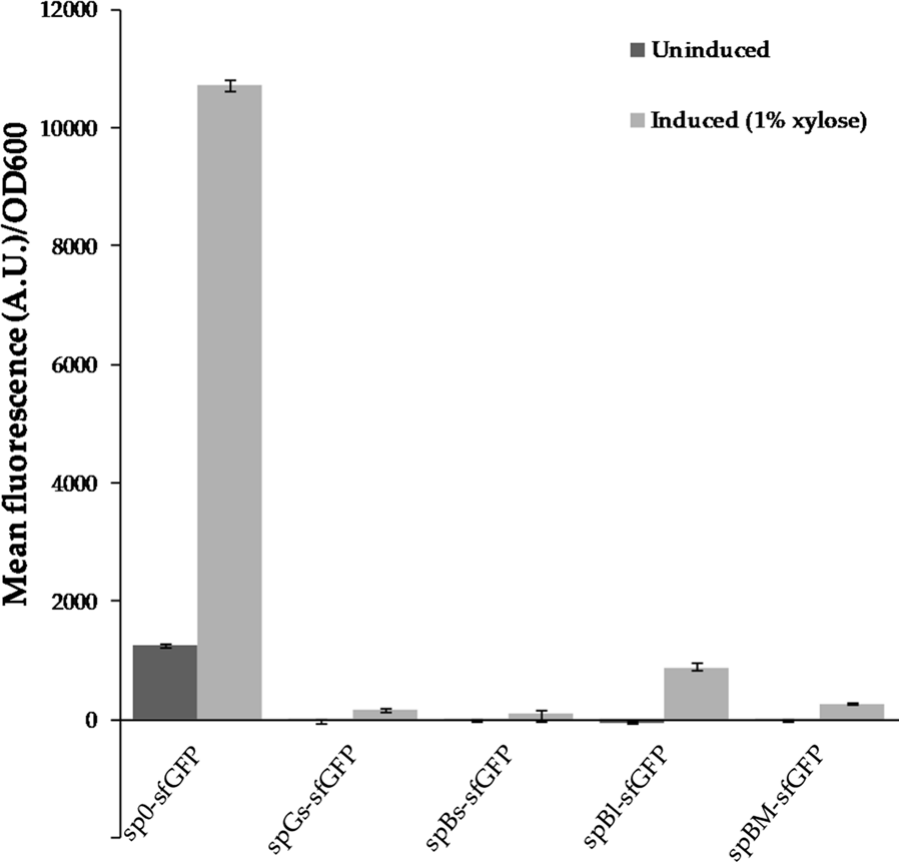
Mean fluorescence intensity (sfGFP) in pellet of *B.* *methanolicus* recombinant strains carrying *sfGFP* gene. The *sfGFP* gene is fused to signal peptides from either G. *stearothermophilus* (strain spGs-sfGFP), *B.* *subtilis* (strain spBs-sfGFP), *B.* *licheniformis* (strain spBl-sfGFP) or *B.* *methanolicus* (strain spBm-sfGFP). Fluorescence from strain carrying vector pBV2xp-*sfGFP* with no signal peptide (sp0-sfGFP) is used as control. Fluorescence from both uninduced (black bars) and induced (grey bars) cultures are indicated

A variant of the GFP, superfolder GFP (sfGFP), folds well when fused to poorly folded polypeptides, and is reported to display increased in vitro thermal stability [82]. The sfGFP-encoding gene was therefore cloned into the pBV2xp vector, and introduced into *B.* *methanolicus*, to determine whether sfGFP could be functionally expressed by *B.* *methanolicus* at 50 °C. As shown in Fig. 4, fluorescence was indeed detected from the pellet of induced cultures of the resulting strain sp0-sfGFP, thereby establishing the first example of a thermostable fluorescent reporter protein being functionally produced by *B.* *methanolicus* at 50 °C. sfGFP was further used as heterologous reporter protein for secretion using the four amylase signal peptides originating from *G.* *stearothermophilus, B.* *subtilis*, *B.* *licheniformis* and *B.* *methanolicus*. The coding sequences of the signal peptides were cloned in-frame with the sfGFP coding sequence in the vector pBV2xp-*sfGFP* and introduced into *B.* *methanolicus* MGA3. The resulting strains, spGs-sfGFP, spBs-sfGFP, spBl-sfGFP and spBm-sfGFP respectively, were cultivated in shake flasks with and without induction by 10 g/L xylose, allowing further characterization of the secretion system. In addition, both supernatants and pellets of the cultivated strains were analyzed for the presence of fluorescence. Weak (notably strain spBl-sfGFP) or no fluorescence could be detected in the pellets of all induced strains carrying signal peptides (Fig. 4; grey bars). This is in contrast to the control strain without a signal peptide (sp0-sfGFP), where strong fluorescence (~ 10,700 units/OD_600_) was detected from the pellet (Fig. 4; grey bars). These results suggested that the strains carrying signal peptides secreted the sfGFP, and this was further investigated below. Without induction of the cultures (Fig. 4; black bars), the fluorescence from the control strain was strongly reduced in comparison to induced conditions (> 85% reduction), however some background expression was still detectable. It has previously been reported that the xylose inducible promoter is not tightly regulated in *B.* *methanolicus*, resulting in background expression [83].

**Fig. 5:**
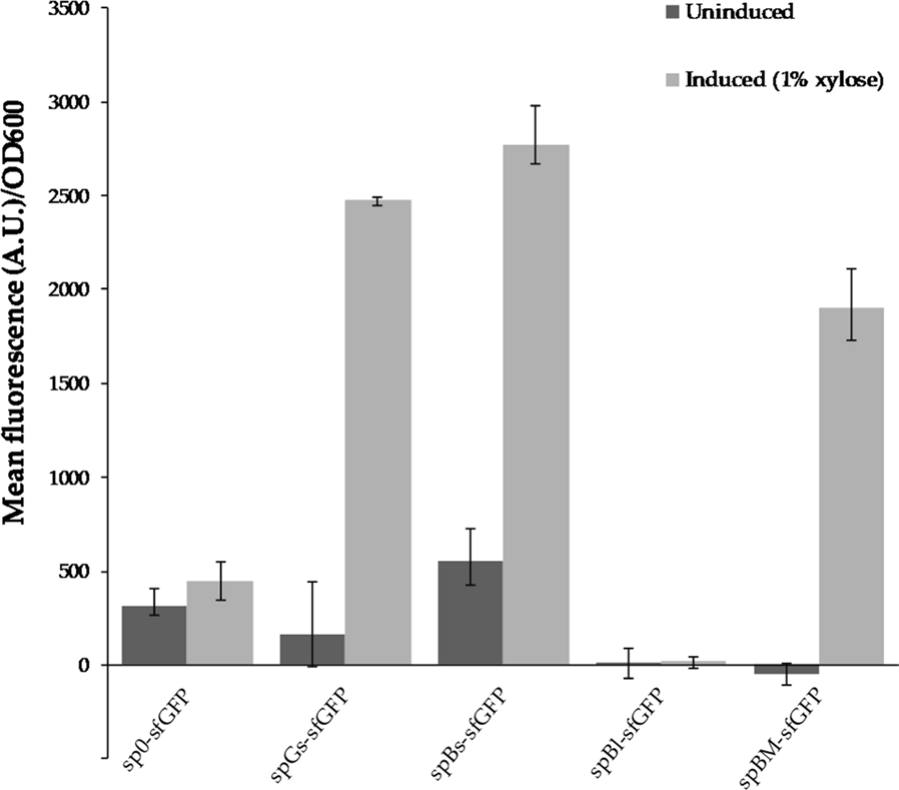
Mean fluorescence intensity (sfGFP) in supernatants of *B.* *methanolicus* recombinant strains carrying *sfGFP* gene. The *sfGFP* gene is fused to signal peptides from α-amylases derived either from *G*. *stearothermophilus* (spGs-sfGFP), *B.* *subtilis* (spBs-sfGFP), *B licheniformis* (spBl-sfGFP) or *B.* *methanolicus* (spBm-sfGFP). Fluorescence from strain carrying vector pBV2xp-sfGFP with no signal peptide (sp0-sfGFP) is used as control. Fluorescence from both uninduced (black bars) and induced (grey bars) cultures are indicated

In the supernatant of the induced cultures, higher fluorescence than from the control strain sp0-sfGFP was detected for all strains, except spBl-sfGFP from which no fluorescence was observed (Fig. 5; grey bars). The functionality of spBl was confirmed with secretion of its native amylase in plate assays, however no secretion could be detected when the peptide was fused to the heterologous reporter protein sfGFP. Interestingly, an apparent fluorescence was detected in the pellets of this strain. This fluorescence was much lower (~ 12-fold) than fluorescence in pellets of the sp0-sfGFP strain. Low total fluorescence (pellet and supernatant) from spBl-sfGFP could be due to low gene expression and improper protein folding or impaired functionality of sfGFP when linked to the *B.* *licheniformis*-derived signal peptide [76, 84]. Furthermore, fusion of signal peptides to sfGFP can result in its thermodynamic destabilization and increased tendency to aggregate [61–63]. Accumulation of intracellular (non-secreted) sfGFP is likely due to a poor signal peptide (spBl)-protein (sfGFP) combination under the conditions tested. The strongest fluorescence intensities were recorded from supernatants of strains spBs-sfGFP and spGs-sfGFP (five to sixfold stronger than the fluorescence intensity from the control strain). It should be noted that some fluorescence was recorded in supernatants of the control strain sp0-sfGFP despite the lack of a signal peptide, and the biological reason for this is unknown. Fluorescence intensity of the supernatants of uninduced cultures of strains carrying signal peptides was similar to or weaker than the fluorescence of the supernatant of the control strain (Fig. 5, black bars). No significant level of sfGFP was retained in the pellets of the secreting strains upon induction, indicating that the secretion machinery of *B.* *methanolicus* was not limiting the secretion of sfGFP under the conditions tested. The presence of sfGFP in the supernatant was subsequently investigated by western blotting.

Western blotting was performed with the supernatants of the induced cultivated strains sp0-sfGFP and spBm-sfGFP (Additional file 1: Figure S4), to confirm that the detected fluorescence in the supernatant was due to production of sfGFP. Total protein concentrations at similar levels were measured in the supernatants of the two strains, and sfGFP was detected in samples from both strains by western blotting. This is in accordance with the fluorescence intensities detected in the supernatants of both tested strains (Fig. 5). Altogether it was shown that functional and controlled protein secretion was achieved using both homologous (spBm) and heterologous (spGs and spBs) signal peptides, and the xylose inducible promoter.

### Recombinant production and secretion of α-amylase enables *B.* *methanolicus* to grow on starch at 50 °C

**Fig. 6:**
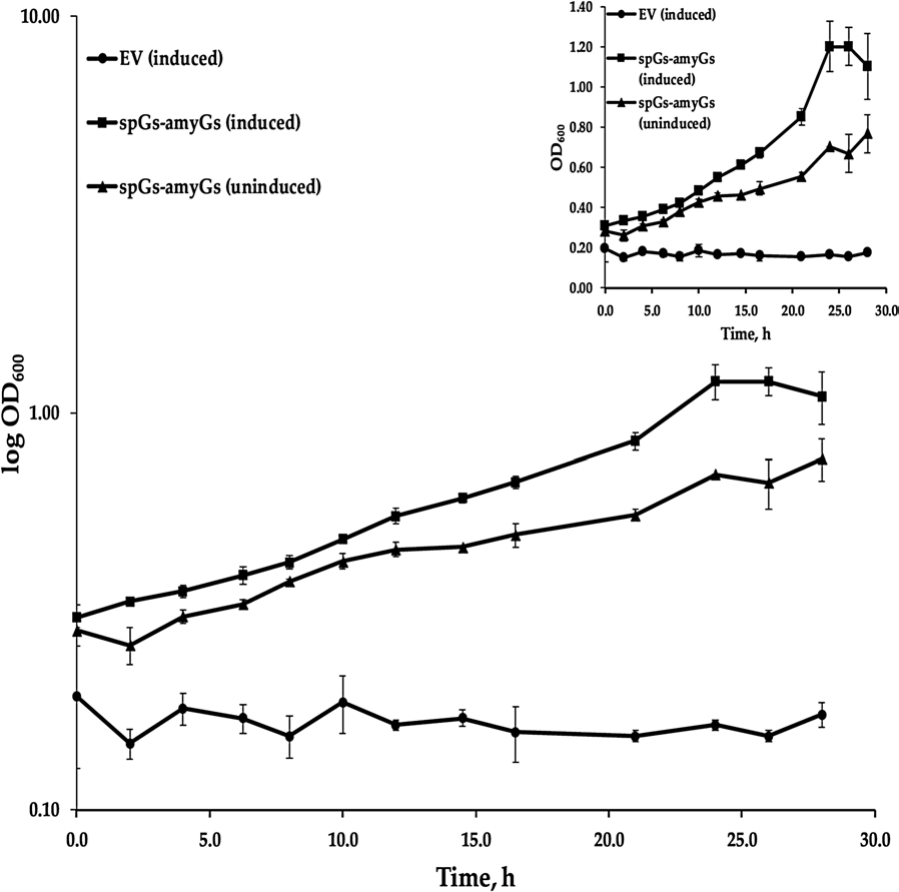
Growth of *B.* *methanolicus* recombinant strains on minimal medium supplemented with starch. spGs-amyGs and the empty vector control strain (EV) were cultivated in minimal medium (MVcM) with 9 g/L soluble starch as carbon source, with or without 10 g/L xylose added as inducer. Maximum and minimum measured values for triplicate cultures are indicated

As a facultative methylotrophic bacterium, *B.* *methanolicus* can utilize a few carbon sources other than methanol for growth, including mannitol and glucose [46–48]. Expanding the range of alternative feedstocks for this bacterium is of interest, especially in terms of polysaccharide utilization. It was therefore compelling to test whether heterologous production and secretion of α-amylase by *B.* *methanolicus* would enable its growth on starch as the sole carbon source. The α-amylase secreting strain spGs-amyGS was selected for this experiment as it exhibited the highest α-amylase activity in the supernatant among the strains tested (Fig. 2). Cultivations of spGs-amyGS and the control strain EV were performed in shake flasks, with minimal medium containing 9 g/L soluble starch as the sole carbon source, with or without 10 g/L xylose for induction. Xylose is neither metabolized by, nor able to support propagation of *B.* *methanolicus* [47, 85]. Growth of the cultures was measured by recording OD_600_ values, as shown in Fig. 6. The induced cultures of strain spGs-amyGS could indeed utilize starch for growth, and this is the first example of growth on starch by *B.* *methanolicus*. Limited cell growth could also be detected for the uninduced cultures of spGs-amyGS, likely caused by background expression of α-amylase from the xylose promoter, as previously published [83] and shown in this work (Figs. 4 and 5). No growth was observed for the strain carrying the empty vector (EV) under the conditions tested, in agreement with results from the α-amylase plate assays (Fig. 1). Maximum OD_600_ values recorded for strain spGs-amyGS were however much lower when starch was used as the carbon source (OD_600_ ≈ 1.2) in comparison to when growing on glucose or methanol (OD_600_ = 7.7 ± 0.4 and 7.7 ± 0.1 respectively) (Additional file 1: Figure S5). Also, the specific growth rate was lower on starch (0.05 ± 0.01 h^−1^) than on glucose (0.16 ± 0.01 h^−1^). Full hydrolyzation of the initially available starch should supply enough glucose to support the same biomass formation as growth on glucose. The concentration of starch in the culture medium was not monitored during growth, however low detected α-amylase activity in liquid assays suggests that the activity of the enzyme could be the limiting factor for growth. The α-amylase activity was highest during methanol-based growth in comparison to growth on starch or glucose for this strain (Additional file 1: Figure S6). The discrepancy in α-amylase activity between two different experiments (cultivated in methanol, Fig. 2 and Additional file 1: Figure S6) is a behaviour previously observed for *B.* *methanolicus* in other studies, for example for acetoin production [83]. Even though the strain was cultivated in the same conditions, the observed α-amylase activities differ over two-fold. This can be caused by the viability of the precultures, the length of the initial lag-phase and other factors. The results were therefore compared within one experiment and not between the two different experiments, in order to avoid drawing false conclusions. Altogether, establishment of protein secretion has enabled functional starch utilization at elevated temperatures in *B.* *methanolicus.*

### Genome-wide in silico prediction of signal peptides in *B.* *methanolicus*

In this work, the secretion of sfGFP was achieved using both native and heterologous signal peptides in *B.* *methanolicus*. To further expand the range of functional signal peptides for secretion of any heterologous protein in this bacterium, an attempt was made to create an in silico signal peptide library. A signal peptide library can serve as a tool in screening studies in order to establish optimal secretion conditions for different heterologous proteins, similarly to previous attempts in different bacterial species [64, 67]. The online server SignalP 4.1 was used to perform genome-wide in silico search for signal peptides belonging to proteins encoded in the genome of *B.* *methanolicus*. Altogether, 3232 protein sequences were analysed, and 169 signal peptides were predicted (Additional file 2: Table S1).

In this work, one of these signal peptides (BMMGA3_04345) has been shown to support secretion of the heterologously produced reporter protein sfGFP at 50 °C. Taking into account that *B.* *methanolicus* in this study was engineered for utilization of a new carbon source (starch), this library might become a valuable source for identifying additional functional signal peptides for secretion of industrially relevant enzymes and for introducing additional complex carbohydrates as carbon sources for this bacterium, thereby expanding its industrial applications.

## Discussion

This work presents the first report on recombinant production and secretion of proteins at 50 °C by the methylotrophic and thermophilic *B.* *methanolicus*. Four genes encoding amylases and three genes encoding proteases, were expressed recombinantly in MGA3 and tested for production of active enzymes in plate assays. Clearing zones were observed for three of the four α-amylase expressing strains tested, spGs-amyGS, spBs-amyBs and spBl-amyBl, upon 24 h incubation on starch at 50 °C. The results indicated that these genes encode active α-amylase proteins with native functional signal peptides. Secretion of proteases by recombinant strains was also confirmed in plate assays, demonstrating that also these proteins can serve as valuable reporter proteins in future studies.

When analyzing supernatants from liquid cultures, secreted amylase activity stronger than the control strain (EV) could be measured for strain spGs-amyGS. No activity higher than that of the control strain (EV) was detected for strains spBs-amyBs and spBl-amyBl, and the reason for the incoherence between plate and liquid assay results is not known. However, the measured α-amylase enzyme activities are close to the detection limit of the assay used in this study. To rule out if the lack of detected activity was solely due to inefficient secretion, the native signal sequences of the latter two genes were replaced with the functional signal peptide spGs from *G.* *stearothermophilus.* The resulting recombinant strains spGs-amyBs and spGs-amyBl produce halos in plate assays, however, analysis of the supernatants showed no detectable amylase activities. These results further indicate that signal peptides are not universal, even within closely related proteins, analogous to previous reports for other bacteria [67]. In our studies the *G.* *stearothermophilus*-derived α-amylase was not tested with alternative signal peptides and therefore it is not known whether the high activity of α-amylase in the supernatant was due to its efficient secretion or expression.

Interestingly, two out of three signal peptides of α-amylases active in plate assay (spGs and spBs), together with spBm, enabled secretion of the heterologous reporter protein sfGFP. However, the ranking of heterologous signal peptides for secretion of sfGFP; spBs, spGs and spBm (in decreasing order), does not completely correspond with the best performing signal peptide for secretion of their native α-amylases (only spGs confirmed in liquid assay). It has previously been reported that different signal peptides have distinct effects on the expression level and secretion efficiency of heterologous proteins, affecting both intracellular and extracellular levels of the indicator proteins [61, 84, 86]. This could also explain the differences in the fluorescence intensity for the different sfGFP secreting strains in our study. As shown in Figs. 4 and 5 (pellet and supernatant), these effects are also visible under non-induced conditions, when using a promoter that is not tightly regulated. As previously shown for *B.* *subtilis*, a single universal signal peptide that will work best for all conditions does not exist [36, 61, 64, 67]. Instead, it has been shown that different proteins require optimized signal peptides, suggesting that a library of different signal peptides is necessary for optimizing secretion of different proteins.

Recombinant expression and secretion of *G.* *stearothermophilus*-derived α-amylase enabled the strain spGs-amyGs to grow on defined medium with starch as the sole carbon source, thus broadening the substrate range of *B.* *methanolicus*. Growth of strain spGs-amyGs on starch (9 g/L) was slower (lower specific growth rate) and led to lower maximum OD_600_ than growth on glucose (9 g/L). The concentration of starch in the medium was not monitored during growth, but the low α-amylase activity observed in previous experiments suggests a low starch hydrolyzation rate, resulting in reduced availability of glucose, thereby directly affecting growth. *B.* *methanolicus* utilizes a mixture of two carbon sources in liquid cultures either concomitantly or sequentially, depending on the available feedstock [48, 87, 88]. By utilizing an inducible promoter to regulate the expression of α-amylase during cultivation on a mixture of starch and another carbon source, the consumption of starch could be controlled, i.e. delaying the utilization of starch until a desired time. In addition, the xylose promoter is titratable in *B. methanolicus*, allowing further control of the expression [54]. The activity of the secreted α-amylase by the spGs-amyGs strain is less than 3 U/mL. Attempts to secrete recombinant proteins in thermophilic conditions by other thermophiles have previously been reported [89–91]. Production of α-amylase in *G. thermoleovorans* resulted in activities of 45.3 U/mL after 12 h at 70 °C [89]. In *Bacillus coagulans* BTS-3, the activity of an extracellular alkaline lipase was 1.16 U/mL in culture supernatant after 48 h at 55 °C [90]. In *Thermus thermophilus,* recombinant Aqualysin I was secreted into the culture medium, with a reported enzyme activity of 600 U/mL after 36 h at 70 °C [91]. In contrast, maximum amylase activities recorded for other *Bacillus* species listed in Wang et al. 2016, are reported to reach over 25,000 U/mL [92]. The low α-amylase activities achieved so far for *B.* *methanolicus* strains in comparison to industrial protein production strains indicate that there is still room for improvements [92]. Some of the possible targets for improvement can be based on the research done for other *Bacillus* species, for example *B.* *subtilis* in which secretion is well studied, as reviewed by Westers et al. [21]. Several potential limitations have been identified, and poor targeting to the translocase machinery (aided by signal peptides), degradation of the secretory protein and incorrect folding are considered among the bottlenecks. Further optimization strategies can include screening for optimal conditions for protein production (pH, temperature, carbon sources), improvement of genetic background (deletion of protease encoding genes, overproduction of chaperones and PrsA), redirection of metabolic flux towards protein production, improvement of the protein secretion system via genetic engineering, and screening of signal peptide libraries for optimal signal peptide-protein fusions [20, 21, 26, 61]. Nonetheless, establishing optimal conditions for production of α-amylase was not within the scope of this work, as the focus was on establishing the necessary tools for production of extracellular proteins in thermophilic conditions.

This work has demonstrated that protein secretion in *B.* *methanolicus* can rely both on native and heterologous signal peptides, presumably with different efficiencies. To enable secretion of any recombinant protein, signal peptide libraries would facilitate selection of optimal signal peptides for each different protein. Hence, a genome-wide analysis of *B. methanolicus* was made in order to create an in silico library of characteristic putative signal peptides. Altogether, 169 putative signal peptides were identified to be encoded in the genome of *B.* *methanolicus.* This number of predicted signal peptides is in accordance with previous analogous studies, where libraries of 173 and 220 signal peptides were established for *B.* *subtilis* and *B.* *licheniformis*, respectively [64]. Signal peptide libraries have earlier been used to establish optimal secretion of different proteins. The *B.* *subtilis*-(native) and *B.* *licheniformis*-derived signal peptide libraries were utilized to optimize secretion of subtilisin BPN’ from *B.* *amyloliquefaciens* in *B.* *subtilis,* and a *B.* *subtilis*-derived signal peptide library was used to establish cutinase secretion in *Corynebacterium glutamicum* [64, 67]. While the intracellular proteome was described for *B.* *methanolicus* by Müller et al. 2014, no analysis of the secretome has been performed to this date, and the in silico prediction presented in this study is consequently not validated with experimental data [52]. However, as already demonstrated in this study, one predicted signal peptide from *B. methanolicus* enabled secretion of the sfGFP reporter protein at 50 °C, supporting the potential of the currently created *B. methanolicus* peptide library.

## Conclusion

The methylotrophic bacterium *Bacillus methanolicus* MGA3 is a promising cell factory for conversion of methanol into value-added products at elevated temperatures. This work demonstrates the first functional production of two types of thermostable reporter proteins, sfGFP and α-amylases, by *B. methanolicus* under thermophilic conditions. The reporter proteins were subsequently used for evaluation and confirmation of the secretion capabilities of *B. methanolicus*, using both homologous and heterologous signal peptides. The potential of *B. methanolicus* as a production host has been expanded by the development of a controlled system for secretion of recombinant proteins at 50 °C, and despite limitations due to low productivity, the possible utility of this system for strain engineering was demonstrated by establishing utilization of starch for growth by *B.* *methanolicus*. Finally, an in silico signal peptide library has been generated, that together with the tools and knowledge presented in this work will be used for further development of *B.* *methanolicus* as a host for recombinant protein production and secretion at 50 °C.

## Methods

### Strains, plasmids and primers

Bacterial strains and plasmids used in this study are listed in Table 2. Abbreviations listed in column two in Table 2 indicate *B.* *methanolicus* strain MGA3 harboring the listed plasmid. Primers are listed in Additional file 2: Table S2.

### Genome-scale and targeted in silico prediction of signal peptides in selected Bacillaceae

The genome-wide prediction of native putative signal peptides encoded in the genome of *B.* *methanolicus* was carried out using the SignalP 4.1 prediction tool for signal peptides characteristic of the Sec pathway with settings for Gram-positive bacteria and default D-cutoff values [93, 94]. Altogether, 3232 genome-encoded protein sequences were analyzed (Accession number: NZ_CP007739.1). Genomes of the closely related species *B.* *subtilis*, *B.* *licheniformis* and *G.* *stearothermophilus* were screened for α-amylase and protease genes with native signal peptides as targets for recombinant secretion in *B.* *methanolicus*. All sequences were analyzed by SignalP 4.1 or 5.0 and PrediSI for determination of signal peptide type and cleavage sites [69, 71, 93, 94].

### Media and growth conditions

*E.* *coli* strains were cultivated at 37 °C in Lysogeny Broth (LB) or on LB agar plates [95] supplemented with either 50 µg/mL kanamycin or 50 µg/mL ampicillin when necessary. For standard cultivations, recombinant strains of *B.* *methanolicus* were cultivated in MVcM minimal medium with 200 mM methanol (MeOH_200_) supplemented with 25 µg/mL kanamycin, unless stated otherwise [96]. For non-methylotrophic conditions, methanol was replaced by either glucose (9 g/L) or soluble starch (9 g/L). When needed, 10 g/L xylose was added for induction. For precultures, minimal medium supplemented with 0.25 g/L yeast extract, designated MVcMY, was used. Cultivations were performed in triplicates in 250 mL baffled flasks (40 mL, 200 rpm, 50 °C), inoculated to a starting OD_600_ = 0.1–0.2. Growth was monitored by measuring OD_600_ with a cell density meter (WPA CO 8000 Biowave). For the EV strain, the OD_600_ values were corrected for the starch background, and for the spGs-AmyGs strain—only for measurements at T0 due to decreasing starch concentration over the growth. Specific growth rates were calculated from the exponential phase, by calculating the slope of semi logarithmic plots of optical density versus time over a suitable time period from an OD_600_ ≥ 0.3. Transformation of *B.* *methanolicus* was performed as previously described [87], with some modifications: After electroporation the cells were cultivated in 5 mL of Super Optimal Broth (SOB) medium at 50 °C for 4–6 h, before plating out on solid SOB medium plates supplemented with 25 µg/mL kanamycin.

### Molecular cloning

Bacterial strains and plasmids constructed and used in this study are listed in Table 1. All cloning work was performed in *Escherichia coli* DH5α. Vectors (when relevant) and all genes were PCR amplified with CloneAmp HiFi PCR mix (TaKaRa Bio), according to the producer’s instructions. Primers used are listed in Additional file 2: Table S2. Plasmid pBV2xp was cut with restriction enzymes SacI and BamHI and joined with PCR-amplified sfGFP or α-amylase- and protease (with their native signal peptides) coding genes by the Gibson assembly reaction [97]. For construction of plasmids harboring α-amylase- and sfGFP-encoding genes with heterologous signal peptides, vectors were PCR amplified as described above and joined with sfGFP- or α-amylase-encoding genes by the Gibson assembly reaction [97].

### α-Amylase and protease plate assays

The recombinant strains to be tested for secretion of α-amylases and proteases were cultivated in 25 mL MeOH_200_ medium for 6–8 h. 5 µL of each culture, diluted to OD_600_ = 1, were placed on SOB plates, supplemented with 5 g/L xylose, 25 µg/mL kanamycin and either 5 g/L starch (α-amylase assay) or 5 g/L skimmed milk (protease assay). The plates were incubated for 12 h at 50 °C followed by incubation at 37 °C or 50 °C for another 12 h. Experiments were performed in duplicates. Degradation of starch by the hydrolyzing α-amylases was visualized by the addition of Lugol’s iodine solution. Colorless halos around cells against the purple background indicated degradation of starch. Clearance of skim milk plates due to proteolytic activity was detected directly by visual observation.

### α-Amylase enzymatic assay, and fluorescence microplate assay

Strains were cultivated at 50 °C in MVcM supplemented with either 200 mM methanol, 9 g/L glucose or 9 g/L starch, and with 25 µg/mL kanamycin and 10 g/L xylose. Strains were harvested when two conditions were met: at least 6 h cultivation time and two doublings were reached. Cells were pelleted by centrifugation (7197 rcf, 10 min, 4 °C). Total protein in supernatants was measured with the Bradford protein assay [98]. Enzymatic activity of α-amylases was measured in culture supernatants at 37 °C, 50 °C and 80 °C by the method of Bernfeld (1955) [99]. For fluorescence microplate assays, pellets were washed twice with PBS, before resuspension in PBS. 200 µL of resuspended cells and/or supernatants were used for measuring fluorescence in microtiter plates (Falcon™ 96-well, clear bottom black polystyrene Imaging Microplate). An Infinite 200Pro plate reader (Tecan Group Ltd.) was used for fluorescence measurements, with settings: ex 485/9 nm, em 535/20 nm. sfGFP signals were collected with a gain setting of 90. Signals were divided by OD_600_ and corrected for background/autofluorescence by subtracting the signal from the strain with an empty plasmid backbone (no signal peptide and no sfGFP).

### Immunoblotting of sfGFP

Induced cell cultures were harvested and proteins in the supernatants were separated by SDS-PAGE. Equal volumes of supernatants were used directly or concentrated using centrifugal filters (Ultracel 3 K). Proteins were blotted onto 0.2 µm PVDF membranes (#1704156) using the Trans-Blot Turbo Transfer System (Bio-Rad) and detection was performed with the iBind system (Invitrogen), using goat anti-GFP polyclonal antibody as the primary antibody (Rockland) and donkey anti-goat IgG (HRP) (Abcam) as the secondary antibody and TMB substrate.

## Supplementary information

**Additional file 1.** Additional Figures S1–S6.

**Additional file 2.** Additional Tables S1, S2.

## Data Availability

The datasets used and/or analyzed during the current study are available from the corresponding author on reasonable request.
