# Supplementary material for: Establishment of a functional system for recombinant production of secreted proteins at 50 °C in the thermophilic Bacillus methanolicus
Source: Microb Cell Fact. 2020 Jul 28;19:151. doi: 10.1186/s12934-020-01409-x (PMC7389648; doi:10.1186/s12934-020-01409-x)
Supplement: Supplementary file 1 — Additional file 1. Additional Figures S1–S6. [file 12934_2020_1409_MOESM1_ESM.docx]

**Additional file 1**


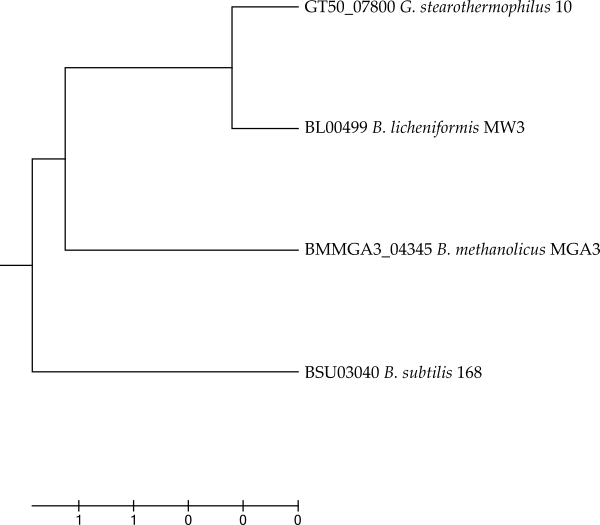


Figure S1 Evolutionary relationships of α-amylases (amino acid) from different Bacillaceae. T**he evolutionary history was inferred using the UPGMA method (1). The optimal tree with the sum of branch length = 3,02966230 is shown. The tree is drawn to scale, with branch lengths in the same units as those of the evolutionary distances used to infer the phylogenetic tree. The evolutionary distances were computed using the Poisson correction method and are in the units of the number of amino acid substitutions per site (2). The analysis involved 4 amino acid sequences. All positions containing gaps and missing data were eliminated. There was a total of 442 positions in the final dataset. Evolutionary analyses were conducted in MEGA7(3)**


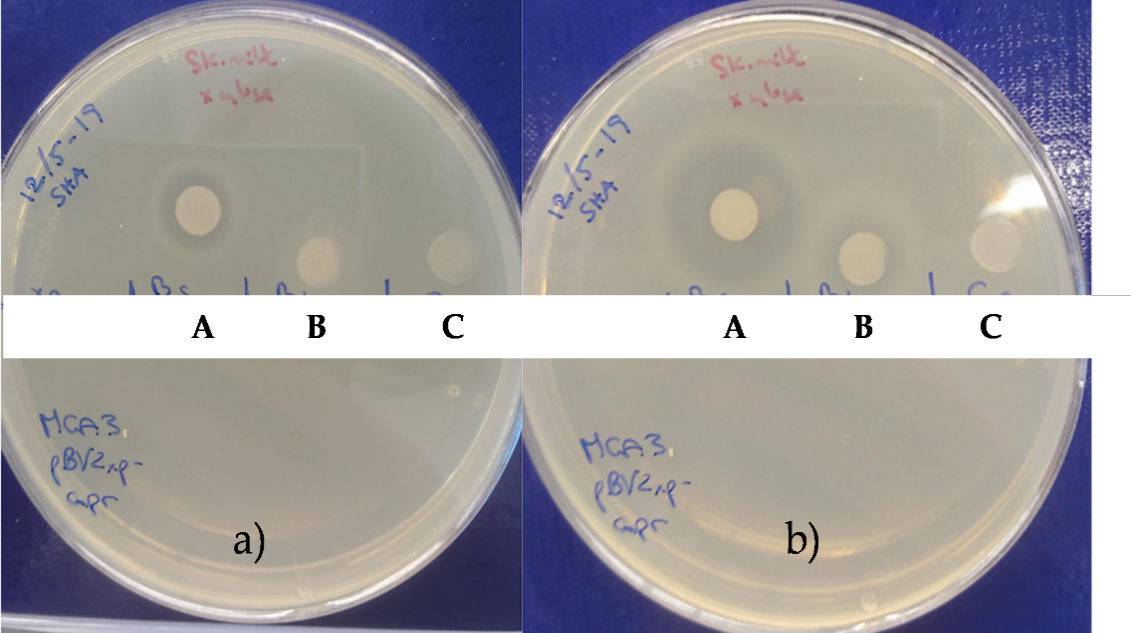


Figure S2 Plate assay for detection of protease activity from recombinant strains of *B.*

*methanolicus* incubated at 50 °C. Degradation of casein by proteases is seen as clearing zones around the

colonies, developed after 24 (a) and 36 (b) hours. Recombinant strains expressing proteases and signal peptides

are: spPBs-aprBs (A), spPBl-aprBl (B) and spPGs-aprGs (C), expressing proteases and signal peptides from either *B. subtilis, B. licheniformis* or *G. stearothermophilus*, respectively.


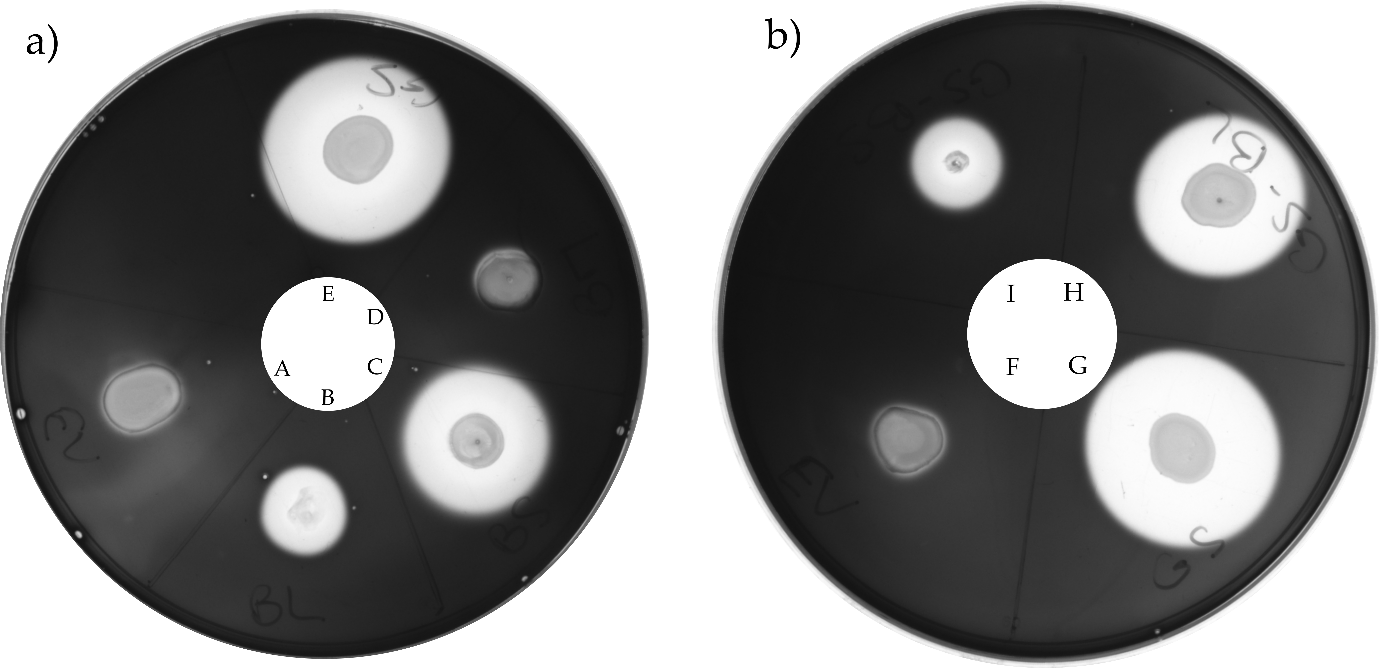


Figure S3 Detection of α-amylase activity from recombinant strains of *B. methanolicus*

incubated at 37 °C. In the plate assay, hydrolysis of starch by α-amylase is seen as clearing zones around the

colonies, visualized by addition of Lugol’s solution after 24 hours incubation. Recombinant strains tested are:

Plate a): EV (emtpy vector), used as control (A), spBl-amyBl (B), spBs-amyBs (C), spBm-amyBm (D) and spGs-amyGs (E). Plate b): Control strain EV (F), spGs-amyGs (G), spGs-amyBl (H) and spGs-amyBs (I).


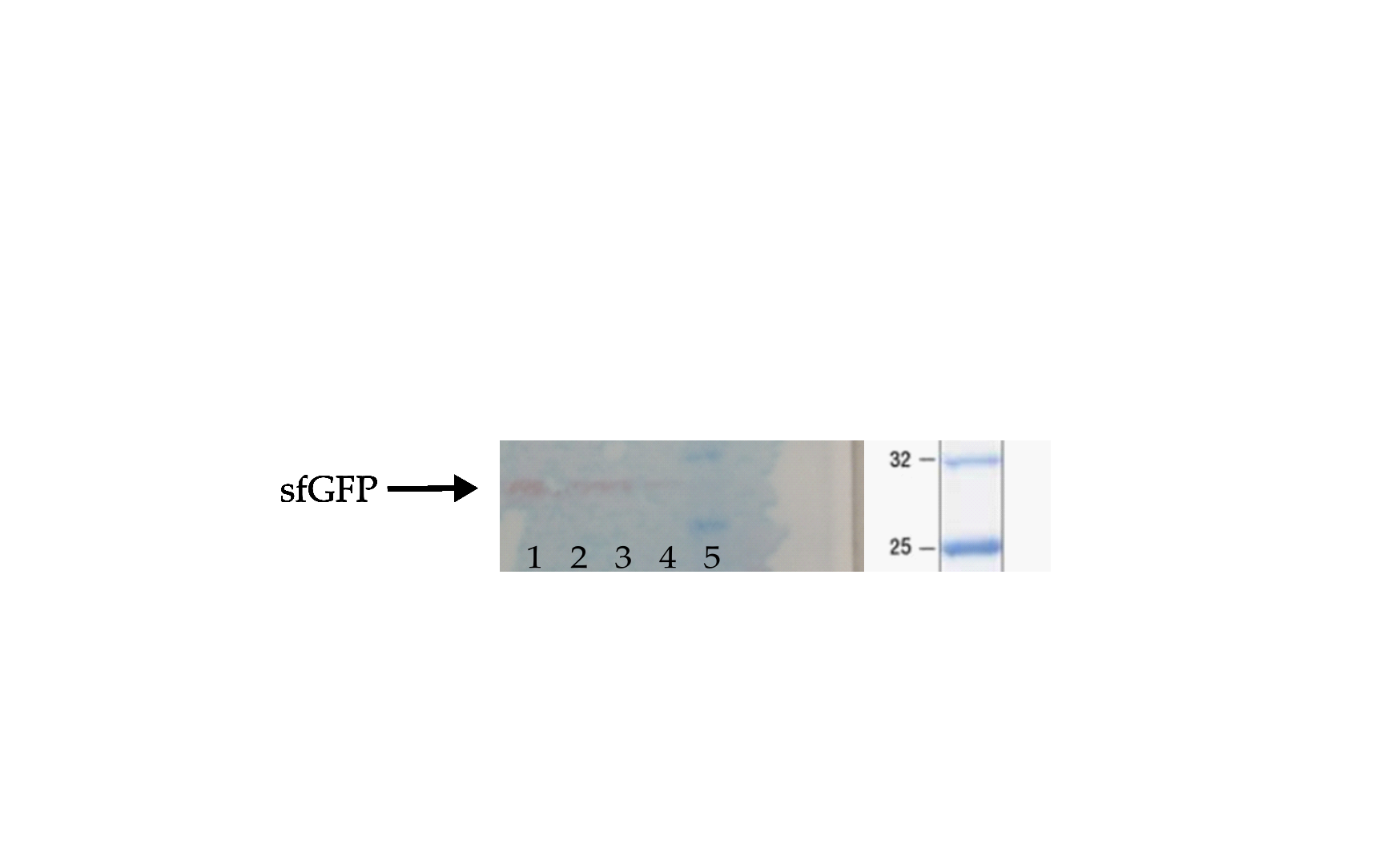


**Figure S4** **Immunoblotting of sfGFP (~27 kDa).** Culture supernatants of spBm-sfGFP and the
control strain, sp0-sfGFP. Samples in lanes 1+2 are concentrated variants of samples in lanes 3+4. From left: 1: spBm-sfGFP, concentrated, 2: sp0-sfGFP, concentrated, 3: spBm-sfGFP, 4: sp0-sfGFP, 5: Protein Standard.


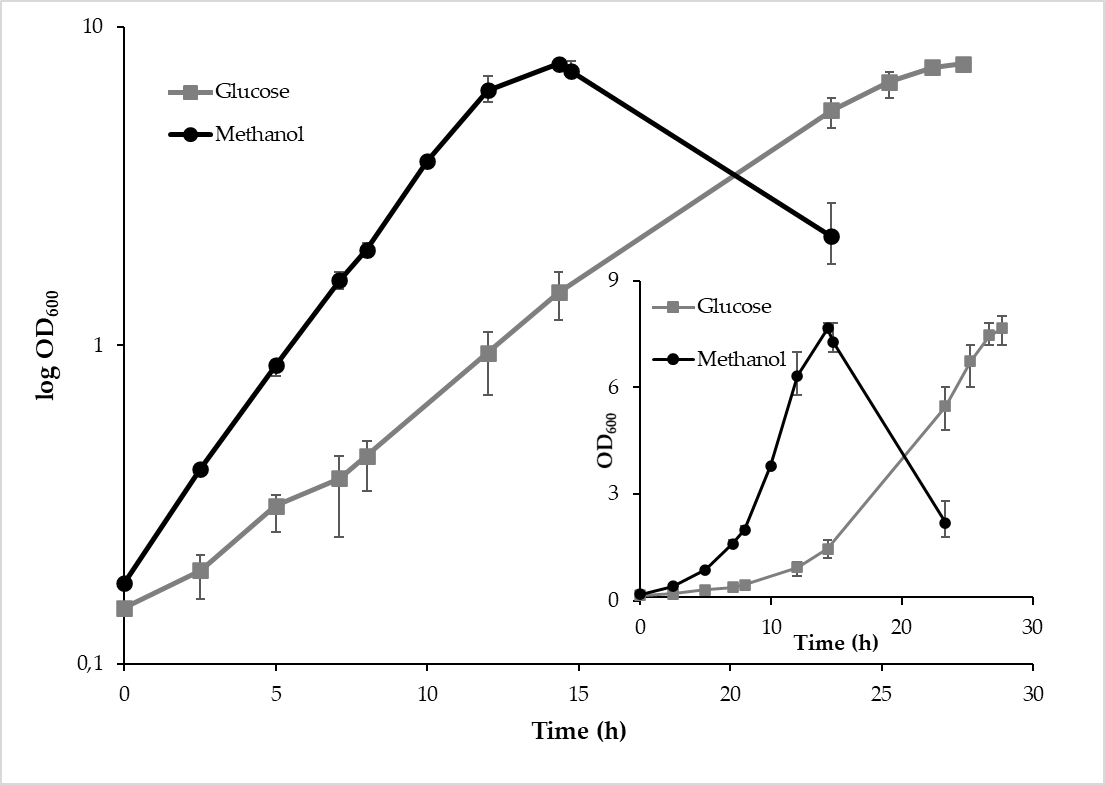
Figure S5. Growth of *B. methanolicus* recombinant strain spGs-amyGs on minimal

medium supplemented with glucose or methanol. spGs-amyGs was cultivated in minimal medium (MVcM)

with 9 g/L glucose or 200 mM methanol as carbon source. Maximum and minimum measured values for

triplicate cultures are indicated. Inset graph shows growth (OD_600_) on linear scale.





**Figure S6. α-Amylase activity in culture supernatants of *B. methanolicus* recombinant strains cultivated in minimal medium supplemented with different carbon sources.** Strains were cultivated in minimial medium (MVcM) with either 200 mM methanol, 0.9% glucose or 0,9% starch as carbon source. Strain spGs-amyGs expresses α-amylase gene from *G. stearothermophilus*. The strain carrying the empty vector (EV) is used as control. Both strains were cultivated in the presence of 1% xylose as inducer. Maximum and minimum measured values for triplicate shake flask cultures are presented.

**Additional material references:**

1. Sneath PH. A, Sokal RR. No Title. Kennedy D, Park RB, editors. Numerical taxonomy, The principles of pratice of nimerical classification. San Francisco: W. H. Freeman and Co; 1973. 573 p.

2. Zuckerkandl E, Pauling L. Evolutionary Divergence and Convergence in Proteins. Evolving Genes and Proteins. 1965.

3. Kumar S, Stecher G, Tamura K. MEGA7: Molecular Evolutionary Genetics Analysis Version 7.0 for Bigger Datasets. Mol Biol Evol. 2016/03/24. 2016;33(7):1870–4.
