# Supplementary material for: Establishment of a functional system for recombinant production of secreted proteins at 50 °C in the thermophilic Bacillus methanolicus
Source: Microb Cell Fact. 2020 Jul 28;19:151. doi: 10.1186/s12934-020-01409-x (PMC7389648; doi:10.1186/s12934-020-01409-x)
Supplement: Supplementary file 2 — Additional file 2. Additional Tables S1, S2. [file 12934_2020_1409_MOESM2_ESM.docx]

Additional file 2

Table S1 Signal peptides predicted by analysis of 3232 protein sequences encoded it the *B. methanolicus* genome. Analyses are performed using SignalP 4.1 (Petersen et al. 2011). LogRPKM values are adapted from the NCBI Gene Expression Omnibus database, accession number GSE64469 (Irla et al. 2015)

| **Gene bank locus tag** | **NCBI Reference sequence locus tag** | **Protein** | **D-score^1^** | **LogRPKM^2^** |
| --- | --- | --- | --- | --- |
| BMMGA3_00075 | BMMGA3_RS00080 | WP_003349802.1 | 0.704 | 173.95 |
| BMMGA3_00240 | BMMGA3_RS00245 | WP_003347138.1 | 0.506 | 1299.79 |
| BMMGA3_00405 | BMMGA3_RS00410 | WP_003347193.1 | 0.484 | 148.37 |
| BMMGA3_00890 | BMMGA3_RS00895 | WP_003348557.1 | 0.51 | 50.74 |
| BMMGA3_01010 | BMMGA3_RS01015 | WP_003348543.1 | 0.567 | 318.15 |
| BMMGA3_01145 | BMMGA3_RS01145 | WP_003348493.1 | 0.466 | 0 |
| BMMGA3_01170 | BMMGA3_RS01170 | WP_003348480.1 | 0.576 | 412.38 |
| BMMGA3_01285 | BMMGA3_RS01285 | WP_034669513.1 | 0.627 | 123.72 |
| BMMGA3_01520 | BMMGA3_RS01525 | WP_034669668.1 | 0.547 | 77.58 |
| BMMGA3_01710 | BMMGA3_RS01715 | WP_003348388.1 | 0.478 | 514.37 |
| BMMGA3_01735 | BMMGA3_RS01740 | WP_003348379.1 | 0.616 | 452.12 |
| BMMGA3_01805 | BMMGA3_RS01810 | WP_003348361.1 | 0.478 | 394.8 |
| BMMGA3_01855 | BMMGA3_RS01860 | WP_003348336.1 | 0.661 | 517.9 |
| BMMGA3_01930 | BMMGA3_RS01920 | WP_003348318.1 | 0.674 | 0 |
| BMMGA3_01955 | BMMGA3_RS01940 | WP_003348311.1 | 0.662 | 395.31 |
| BMMGA3_01960 | BMMGA3_RS16660 | WP_003348309.1 | 0.561 | 290.28 |
| BMMGA3_01980 | BMMGA3_RS01965 | WP_003348298.1 | 0.517 | 181.4 |
| BMMGA3_02005 | BMMGA3_RS01990 | WP_003348288.1 | 0.765 | 0 |
| BMMGA3_02035 | BMMGA3_RS02020 | WP_003348276.1 | 0.572 | 772.97 |
| BMMGA3_02065 | BMMGA3_RS02050 | WP_038501993.1 | 0.686 | 98.39 |
| BMMGA3_02145 | BMMGA3_RS02130 | WP_081485694.1 | 0.458 | 7.01 |
| BMMGA3_02225 | BMMGA3_RS02200 | WP_003348208.1 | 0.704 | 101.09 |
| BMMGA3_02230 | BMMGA3_RS02205 | WP_003348206.1 | 0.75 | 80.24 |
| BMMGA3_02250 | BMMGA3_RS02220 | WP_003348200.1 | 0.693 | 0 |
| BMMGA3_02280 | BMMGA3_RS02255 | WP_003348191.1 | 0.623 | 0 |
| BMMGA3_02310 | BMMGA3_RS16685 | WP_003348178.1 | 0.776 | 0 |
| BMMGA3_02345 | BMMGA3_RS02320 | WP_003348170.1 | 0.805 | 131.15 |
| BMMGA3_02415 | BMMGA3_RS02385 | WP_003348139.1 | 0.573 | 0 |
| BMMGA3_02430 | BMMGA3_RS02400 | WP_003348136.1 | 0.527 | 0 |
| BMMGA3_02600 | BMMGA3_RS02570 | WP_003348070.1 | 0.766 | 194.18 |
| BMMGA3_02715 | BMMGA3_RS02675 | WP_003348033.1 | 0.595 | 192.62 |
| BMMGA3_02830 | BMMGA3_RS02785 | WP_003348000.1 | 0.73 | 178.85 |
| BMMGA3_02860 | BMMGA3_RS02815 | WP_003347993.1 | 0.602 | 413.34 |
| BMMGA3_02895 | BMMGA3_RS02845 | WP_003347980.1 | 0.639 | 0 |
| BMMGA3_03295 | BMMGA3_RS03215 | WP_034669491.1 | 0.724 | 0 |
| BMMGA3_03310 | BMMGA3_RS03230 | WP_003347829.1 | 0.543 | 0 |
| BMMGA3_03350 | BMMGA3_RS03270 | WP_003347807.1 | 0.743 | 28.53 |
| BMMGA3_03470 | BMMGA3_RS03380 | WP_003347767.1 | 0.808 | 86.37 |
| BMMGA3_03665 | BMMGA3_RS03570 | WP_004433240.1 | 0.632 | 10.82 |
| BMMGA3_03775 | BMMGA3_RS03675 | WP_004433278.1 | 0.458 | 18.36 |
| BMMGA3_03795 | BMMGA3_RS03695 | WP_004433285.1 | 0.626 | 23.13 |
| BMMGA3_04185 | BMMGA3_RS04100 | WP_004433451.1 | 0.676 | 199.84 |
| BMMGA3_04345 | BMMGA3_RS04265 | WP_004433531.1 | 0.528 | 128.81 |
| BMMGA3_04375 | BMMGA3_RS04295 | WP_004433544.1 | 0.555 | 0 |
| BMMGA3_04515 | BMMGA3_RS04430 | WP_004433603.1 | 0.66 | 831.27 |
| BMMGA3_04635 | BMMGA3_RS04550 | WP_004433644.1 | 0.545 | 0 |
| BMMGA3_04690 | BMMGA3_RS04600 | WP_004433659.1 | 0.635 | 148.54 |
| BMMGA3_04835 | BMMGA3_RS04710 | WP_034669198.1 | 0.512 | 29.8 |
| BMMGA3_05120 | BMMGA3_RS04960 | WP_003348704.1 | 0.585 | 83.31 |
| BMMGA3_05270 | BMMGA3_RS05110 | WP_003348757.1 | 0.509 | 751.35 |
| BMMGA3_05405 | BMMGA3_RS05230 | WP_003348797.1 | 0.622 | 0 |
| BMMGA3_05550 | BMMGA3_RS05375 | WP_003348850.1 | 0.536 | 0 |
| BMMGA3_05660 | BMMGA3_RS05485 | WP_003348886.1 | 0.48 | 576.5 |
| BMMGA3_06160 | BMMGA3_RS05990 | WP_003349058.1 | 0.574 | 517.02 |
| BMMGA3_06180 | BMMGA3_RS06010 | WP_003349066.1 | 0.782 | 145.97 |
| BMMGA3_06425 | BMMGA3_RS06260 | WP_003349142.1 | 0.506 | 330.6 |
| BMMGA3_06655 | BMMGA3_RS06480 | WP_003349220.1 | 0.456 | 7.11 |
| BMMGA3_06870 | BMMGA3_RS06675 | WP_003349285.1 | 0.49 | 0 |
| BMMGA3_06950 | BMMGA3_RS06750 | WP_003349314.1 | 0.797 | 0 |
| BMMGA3_06970 | BMMGA3_RS06770 | WP_003349318.1 | 0.5 | 16.62 |
| BMMGA3_07075 | BMMGA3_RS06870 | WP_003349613.1 | 0.455 | 0 |
| BMMGA3_07365 | BMMGA3_RS07135 | WP_004433795.1 | 0.601 | 80.53 |
| BMMGA3_07590 | BMMGA3_RS07365 | WP_004433812.1 | 0.713 | 40.84 |
| BMMGA3_07630 | BMMGA3_RS07405 | WP_004433827.1 | 0.532 | 113.84 |
| BMMGA3_07705 | BMMGA3_RS07465 | WP_034669201.1 | 0.52 | 0 |
| BMMGA3_07865 | BMMGA3_RS07600 | WP_004433904.1 | 0.655 | 0 |
| BMMGA3_07950 | BMMGA3_RS07680 | WP_004433934.1 | 0.612 | 44.69 |
| BMMGA3_08235 | BMMGA3_RS07955 | WP_004434024.1 | 0.488 | 71.76 |
| BMMGA3_08360 | BMMGA3_RS08070 | WP_004434093.1 | 0.576 | 159.1 |
| BMMGA3_08370 | BMMGA3_RS08080 | WP_004434100.1 | 0.518 | 0 |
| BMMGA3_08395 | BMMGA3_RS08105 | WP_004434115.1 | 0.668 | 32.75 |
| BMMGA3_08410 | BMMGA3_RS08120 | WP_034669333.1 | 0.536 | 77.17 |
| BMMGA3_08515 | BMMGA3_RS08210 | WP_004434174.1 | 0.549 | 73.6 |
| BMMGA3_08560 | BMMGA3_RS08255 | WP_004434205.1 | 0.517 | 166.26 |
| BMMGA3_08590 | BMMGA3_RS08285 | WP_004434218.1 | 0.499 | 57.14 |
| BMMGA3_08620 | BMMGA3_RS08315 | WP_004434237.1 | 0.479 | 42 |
| BMMGA3_08730 | BMMGA3_RS08415 | WP_034669206.1 | 0.591 | 183.51 |
| BMMGA3_08755 | BMMGA3_RS08440 | WP_004434308.1 | 0.498 | 52.89 |
| BMMGA3_08775 | BMMGA3_RS08460 | WP_004434315.1 | 0.501 | 314.73 |
| BMMGA3_08820 | BMMGA3_RS08505 | WP_004434344.1 | 0.657 | 289.8 |
| BMMGA3_08880 | BMMGA3_RS08560 | WP_004434368.1 | 0.665 | 36.99 |
| BMMGA3_08885 | BMMGA3_RS08565 | WP_004434370.1 | 0.501 | 151.68 |
| BMMGA3_08925 | BMMGA3_RS08605 | WP_004434388.1 | 0.623 | 298.9 |
| BMMGA3_08935 | BMMGA3_RS08615 | WP_004434392.1 | 0.476 | 485.69 |
| BMMGA3_08980 | BMMGA3_RS08660 | WP_004434405.1 | 0.489 | 172.88 |
| BMMGA3_09105 | BMMGA3_RS08780 | WP_004434447.1 | 0.669 | 52.12 |
| BMMGA3_09245 | BMMGA3_RS16795 | WP_004434511.1 | 0.691 | 950.28 |
| BMMGA3_09250 | BMMGA3_RS08915 | WP_004434514.1 | 0.684 | 117.68 |
| BMMGA3_09325 | BMMGA3_RS08985 | WP_004434562.1 | 0.722 | 13.05 |
| BMMGA3_09330 | BMMGA3_RS16800 | WP_004434563.1 | 0.602 | 381.3 |
| BMMGA3_09470 | BMMGA3_RS09125 | WP_004434641.1 | 0.783 | 286.99 |
| BMMGA3_09910 | BMMGA3_RS09520 | WP_004434872.1 | 0.717 | 0 |
| BMMGA3_10020 | BMMGA3_RS09625 | WP_004434934.1 | 0.689 | 24.97 |
| BMMGA3_10035 | BMMGA3_RS09640 | WP_004434942.1 | 0.598 | 37.02 |
| BMMGA3_10050 | BMMGA3_RS09655 | WP_004434953.1 | 0.686 | 173.04 |
| BMMGA3_10065 | BMMGA3_RS09670 | WP_004434970.1 | 0.496 | 12 |
| BMMGA3_10075 | BMMGA3_RS09680 | WP_004434972.1 | 0.478 | 80.45 |
| BMMGA3_10210 | BMMGA3_RS09795 | WP_004435024.1 | 0.547 | 113.4 |
| BMMGA3_10250 | BMMGA3_RS09830 | WP_004435041.1 | 0.46 | 36.69 |
| BMMGA3_10260 | BMMGA3_RS09840 | WP_004435043.1 | 0.538 | 67.59 |
| BMMGA3_10395 | BMMGA3_RS09975 | WP_004435114.1 | 0.551 | 41.9 |
| BMMGA3_10670 | BMMGA3_RS10235 | WP_004435257.1 | 0.747 | 0 |
| BMMGA3_10795 | BMMGA3_RS10360 | WP_004435301.1 | 0.589 | 143.77 |
| BMMGA3_10875 | BMMGA3_RS10435 | WP_034669244.1 | 0.569 | 33.59 |
| BMMGA3_10955 | BMMGA3_RS10515 | WP_004435360.1 | 0.484 | 0 |
| BMMGA3_11010 | BMMGA3_RS10570 | WP_004435382.1 | 0.465 | 25.5 |
| BMMGA3_11085 | BMMGA3_RS10645 | WP_004435415.1 | 0.81 | 23.28 |
| BMMGA3_11240 | BMMGA3_RS10795 | WP_004435491.1 | 0.765 | 320.89 |
| BMMGA3_11285 | BMMGA3_RS10845 | WP_004435514.1 | 0.546 | 150.79 |
| BMMGA3_11450 | BMMGA3_RS11010 | WP_004435575.1 | 0.706 | 0 |
| BMMGA3_11765 | BMMGA3_RS11320 | WP_004435717.1 | 0.46 | 0 |
| BMMGA3_12055 | BMMGA3_RS11600 | WP_004435837.1 | 0.53 | 57.02 |
| BMMGA3_12320 | BMMGA3_RS11865 | WP_004435970.1 | 0.662 | 98.3 |
| BMMGA3_12430 | BMMGA3_RS11975 | WP_003347686.1 | 0.605 | 105.95 |
| BMMGA3_12610 | BMMGA3_RS12150 | WP_003347624.1 | 0.69 | 63.43 |
| BMMGA3_12640 | BMMGA3_RS12180 | WP_003347611.1 | 0.611 | 143.95 |
| BMMGA3_12830 | BMMGA3_RS12375 | WP_003347538.1 | 0.468 | 80.44 |
| BMMGA3_13495 | BMMGA3_RS13040 | WP_003347303.1 | 0.664 | 79.51 |
| BMMGA3_13730 | BMMGA3_RS13265 | WP_003347219.1 | 0.488 | 405.57 |
| BMMGA3_13965 | BMMGA3_RS13500 | WP_003349790.1 | 0.451 | 0 |
| BMMGA3_14150 | BMMGA3_RS13675 | WP_003349564.1 | 0.632 | 50.78 |
| BMMGA3_14200 | BMMGA3_RS13725 | WP_003349553.1 | 0.731 | 55.36 |
| BMMGA3_14270 | BMMGA3_RS13795 | WP_003349530.1 | 0.58 | 25.5 |
| BMMGA3_14280 | BMMGA3_RS13805 | WP_003349527.1 | 0.723 | 0 |
| BMMGA3_14285 | BMMGA3_RS13810 | WP_003349525.1 | 0.564 | 63.19 |
| BMMGA3_14365 | BMMGA3_RS13885 | WP_003349503.1 | 0.704 | 751.22 |
| BMMGA3_14420 | BMMGA3_RS17585 | WP_081485711.1 | 0.498 | 0 |
| BMMGA3_14495 | BMMGA3_RS14010 | WP_003349465.1 | 0.753 | 18.18 |
| BMMGA3_14500 | BMMGA3_RS14015 | WP_003349464.1 | 0.488 | 0 |
| BMMGA3_14805 | BMMGA3_RS14310 | WP_003349381.1 | 0.748 | 0 |
| BMMGA3_14880 | BMMGA3_RS14385 | WP_038502312.1 | 0.486 | 30.37 |
| BMMGA3_14890 | BMMGA3_RS14400 | WP_003346411.1 | 0.707 | 397.39 |
| BMMGA3_14905 | BMMGA3_RS14415 | WP_003346415.1 | 0.609 | 476.69 |
| BMMGA3_15055 | BMMGA3_RS14570 | WP_034669139.1 | 0.504 | 226.18 |
| BMMGA3_15060 | BMMGA3_RS14575 | WP_003346484.1 | 0.754 | 83.66 |
| BMMGA3_15065 | BMMGA3_RS14580 | WP_003346486.1 | 0.48 | 123.84 |
| BMMGA3_15070 | BMMGA3_RS14585 | WP_003346488.1 | 0.857 | 504.25 |
| BMMGA3_15150 | BMMGA3_RS14665 | WP_003346515.1 | 0.82 | 157.74 |
| BMMGA3_15160 | BMMGA3_RS14675 | WP_003346517.1 | 0.502 | 202.31 |
| BMMGA3_15165 | BMMGA3_RS16815 | WP_081848756.1 | 0.641 | 247.2 |
| BMMGA3_15185 | BMMGA3_RS14700 | WP_003346523.1 | 0.737 | 111.44 |
| BMMGA3_15195 | BMMGA3_RS14710 | WP_003346526.1 | 0.647 | 260.33 |
| BMMGA3_15275 | BMMGA3_RS14790 | WP_003346559.1 | 0.592 | 8.97 |
| BMMGA3_15345 | BMMGA3_RS14850 | WP_003346585.1 | 0.724 | 0 |
| BMMGA3_15380 | BMMGA3_RS14885 | WP_003346599.1 | 0.805 | 288.91 |
| BMMGA3_15450 | BMMGA3_RS14955 | WP_003346617.1 | 0.56 | 24.58 |
| BMMGA3_15470 | BMMGA3_RS16820 | WP_003346618.1 | 0.827 | 133.36 |
| BMMGA3_15480 | BMMGA3_RS16825 | WP_003346621.1 | 0.856 | 540.14 |
| BMMGA3_15485 | BMMGA3_RS14980 | WP_003346623.1 | 0.657 | 69.31 |
| BMMGA3_15565 | BMMGA3_RS15055 | WP_003346653.1 | 0.497 | 71.03 |
| BMMGA3_15600 | BMMGA3_RS15090 | WP_003346669.1 | 0.719 | 313.83 |
| BMMGA3_15605 | BMMGA3_RS15095 | WP_003346670.1 | 0.561 | 321.09 |
| BMMGA3_15610 | BMMGA3_RS16835 | WP_003346672.1 | 0.836 | 199.72 |
| BMMGA3_15635 | BMMGA3_RS16840 | WP_003346679.1 | 0.744 | 109.48 |
| BMMGA3_15720 | BMMGA3_RS15200 | WP_003346702.1 | 0.49 | 134.31 |
| BMMGA3_15725 | BMMGA3_RS15205 | WP_003346704.1 | 0.635 | 16.25 |
| BMMGA3_15730 | BMMGA3_RS16845 | WP_003346706.1 | 0.575 | 7.59 |
| BMMGA3_15740 | BMMGA3_RS16850 | WP_003346714.1 | 0.694 | 976.29 |
| BMMGA3_15745 | BMMGA3_RS15225 | WP_003346719.1 | 0.734 | 63.19 |
| BMMGA3_15750 | BMMGA3_RS15230 | WP_003346720.1 | 0.756 | 26.69 |
| BMMGA3_15755 | BMMGA3_RS15235 | WP_003346723.1 | 0.672 | 33.12 |
| BMMGA3_15780 | BMMGA3_RS15250 | WP_003346727.1 | 0.644 | 80.67 |
| BMMGA3_15835 | BMMGA3_RS15305 | WP_003346753.1 | 0.648 | 290.16 |
| BMMGA3_15900 | BMMGA3_RS15365 | WP_003346773.1 | 0.578 | 77.81 |
| BMMGA3_16025 | BMMGA3_RS15490 | WP_003346816.1 | 0.556 | 14.33 |
| BMMGA3_16060 | BMMGA3_RS15525 | WP_003346833.1 | 0.47 | 137.11 |
| BMMGA3_16345 | BMMGA3_RS15805 | WP_003346929.1 | 0.567 | 207.52 |
| BMMGA3_16500 | BMMGA3_RS15950 | WP_003346994.1 | 0.462 | 0 |
| BMMGA3_16530 | BMMGA3_RS15980 | WP_003347005.1 | 0.678 | 107.43 |

^1^ D-score (discrimination score): A weighted average of the mean S and the maximal Y scores where C-score is the predicted cleavage site value, S-score is the predicted signal peptide value and Y-score is a combination of C- and S-scores. D-score the score that is used to discriminate signal peptides from non-signal peptides, as described in Nielsen 2017.
^2^Log RPKM: log reads per kilobase million. Value used to normalize RNA-seq data, and here used for comparison of transcript abundancies, as described in Irla et al. 2015.

Table S2 Primers used in this study

| **Name** | **Sequence** | **Length** | **Comment** |
| --- | --- | --- | --- |
| PS00a | TTCACTTAAGGGGGAAATGGCAAATGCGTAAAGGCGAAGAGCTGT | 45 | FW sfGFP for insertion into pBV2xp |
| PS00b | ACGGCCAGTGAATTCGAGCTTCATTTGTACAGTTCATCCATAC | 43 | RW sfGFP for insertion into pBV2xp |
| PS01 | TTCACTTAAGGGGGAAATGGCAAATGATGAGGAAAAAGAGTTTTTGGCTTGGGATG | 56 | FW *apr* *B. licheniformis* ATCC14580 |
| PS02 | ACGACGGCCAGTGAATTCGAGCTTTATTGAGCGGCAGCTTCGACATTGAT | 50 | RW *apr* *B. licheniformis* ATCC14580 |
| PS03 | TTCACTTAAGGGGGAAATGGCAAATGAGAAGCAAAAAATTGTGGATCAGCTTGTTG | 56 | FW *apr* *B. subtilis* 168 |
| PS04 | ACGACGGCCAGTGAATTCGAGCTTTATTGTGCAGCTGCTTGTACGTTGATTAAC | 54 | RW *apr* *B. subtilis* 168 |
| PS05 | TTCACTTAAGGGGGAAATGGCAAATGAAACGCCGATACCGA | 41 | FW *apr* *G. stearothermophilus* 10 |
| PS06 | ACGACGGCCAGTGAATTCGAGCTTTATCGCTTAGCCAGCTC | 41 | RW *apr* *G. stearothermophilus* 10 |
| PS07 | TTCACTTAAGGGGGAAATGGCAAATGAAACAACAAAAACGGCTTTACGCCCGAT | 54 | FW *amyL* *B. licheniformis* ATCC14580 |
| PS08 | ACGACGGCCAGTGAATTCGAGCTCTATCTTTGAACATAAATTGAAACCGACCCGCCGTTTAC | 62 | FW *amyL* *B. licheniformis* ATCC14580 |
| PS09 | TTCACTTAAGGGGGAAATGGCAAATGTTTGCAAAACGATTCAAAACCTCTTTACTGCCGTTAT | 63 | FW *amyE* *B. subtilis* 168 |
| PS10 | ACGACGGCCAGTGAATTCGAGCTTCAATGGGGAAGAGAACCGCTTAA | 47 | FW *amyE* *B. subtilis* 168 |
| PS11 | TTCACTTAAGGGGGAAATGGCAAATGCTAACGTTTCACCGCATCA | 45 | FW *amyS* *G. stearothermophilus* 10 |
| PS12 | ACGACGGCCAGTGAATTCGAGCTTCAAGGCCATGCCACCAA | 41 | RW *amyS* *G. stearothermophilus* 10 |
| PS13 | TTCACTTAAGGGGGAAATGGCAAATGAAAAAATTTTTTCTTACTTTCATCTTAATCCCGTTTC | 63 | FW *amy* *B.  methanolicus* MGA3 |
| PS14 | ACGACGGCCAGTGAATTCGAGCTCTAGGAGATTTTTTTTCTTCCTTTC | 48 | RW *amy* *B. methanolicus* MGA3 |
| PS26 | GCGAATGGCGCTAGAGCTTGGCACTG | 26 | FW pBV2xp, for amplification of vector backbone with signal peptide |
| PS27 | AGCCTTGGCGGGCTGTCCTGTTG | 23 | RW Backbone (pBV2xp) + spGS |
| PS28 | CGCCGCTGCTGCAGAATGAG | 20 | RW Backbone + spBl |
| PS29 | AGCACTCGCAGCCGCCGGTCCTGCCAGAAC | 30 | RW Backbone + spBs |
| PS30 | TGCTGCCTCTATTGGGGCGGAGT | 23 | RW Backbone + spBm |
| PS31 | CAAGCTCTAGCGCCATTCGCTCATTTGTACAGTTCATCCATACCATGCGTGATGC | 55 | RW *sfGFP* |
| PS32 | ACAGGACAGCCCGCCAAGGCTATGCGTAAAGGCGAAGAGCTGTTCACT | 48 | FW spGs-*sfGFP* |
| PS33 | CTCATTCTGCAGCAGCGGCGATGCGTAAAGGCGAAGAGCTGTTCACT | 47 | FW spBl-*sfGFP* |
| PS34 | AGGACCGGCGGCTGCGAGTGCTATGCGTAAAGGCGAAGAGCTGTTCACT | 49 | FW spBs-*sfGFP* |
| PS35 | TCCGCCCCAATAGAGGCAGCAATGCGTAAAGGCGAAGAGCTGTTCACT | 48 | FW spBm-*sfGFP* |
| PS52 | ACAGGACAGCCCGCCAAGGCTGCAAATCTTAAAGGGACGCTGATGCAGTATTTTGA | 56 | FW *amyL* for spGs |
| PS53 | CAAGCTCTAGCGCCATTCGCCTATCTTTGAACATAAATTGAAACCGACCCGCCGTTTAC | 59 | RW *amyL* and *amyE* for spGs |
| PS54 | ACAGGACAGCCCGCCAAGGCTGAAACGGCGAACAAATCG | 39 | FW *amyE* for spGs |
